# The Changes of qEEG Approximate Entropy during Test of Variables of Attention as a Predictor of Major Depressive Disorder

**DOI:** 10.3390/brainsci10110828

**Published:** 2020-11-07

**Authors:** Shao-Tsu Chen, Li-Chi Ku, Shaw-Ji Chen, Tsu-Wang Shen

**Affiliations:** 1 Department of Psychiatry, Hualien Tzu Chi Hospital, Buddhist Tzu-Chi Medical Foundation, Hualien 970, Taiwan; shaotsu.tw@yahoo.com.tw; 2 Department of Psychiatry, Tzu Chi University, Hualien 970, Taiwan; 3 Department of Medical Informatics, Tzu Chi University, Hualien 970, Taiwan; joohrikkb@gmail.com; 4 Department of Psychiatry, Taitung MacKay Memorial Hospital, Taitung County 950, Taiwan; shawjichen@hotmail.com; 5 Department of Medicine, MacKay Medical College, New Taipei City 252, Taiwan; 6 Department of Automatic Control Engineering, Feng Chia University, Taichung 40724, Taiwan; 7 Master’s Program Biomedical Informatics and Biomedical Engineering, Feng Chia University, Taichung 40724, Taiwan

**Keywords:** quantitative electroencephalography, test of variables of attention, major depressive disorder, approximate entropy, cordance

## Abstract

Evaluating brain function through biosignals remains challenging. Quantitative electroencephalography (qEEG) outcomes have emerged as a potential intermediate biomarker for diagnostic clarification in psychological disorders. The Test of Variables of Attention (TOVA) was combined with qEEG to evaluate biomarkers such as absolute power, relative power, cordance, and approximate entropy from covariance matrix images to predict major depressive disorder (MDD). EEG data from 18 healthy control and 18 MDD patients were monitored during the resting state and TOVA. TOVA was found to provide aspects for the evaluation of MDD beyond resting electroencephalography. The results showed that the prefrontal qEEG theta cordance of the control and MDD groups were significantly different. For comparison, the changes in qEEG approximate entropy (ApEn) patterns observed during TOVA provided features to distinguish between participants with or without MDD. Moreover, ApEn scores during TOVA were a strong predictor of MDD, and the ApEn scores correlated with the Beck Depression Inventory (BDI) scores. Between-group differences in ApEn were more significant for the testing state than for the resting state. Our results provide further understanding for MDD treatment selection and response prediction during TOVA.

## 1. Introduction

Depression is a common psychiatric disorder; however, to quantify depressive disorder through biosignals remains challenging. According to a study from the National Institute of Mental Health, depression onset may occur at any age. The World Health Organization reports that more than 264 million people of all ages have depression worldwide [1]. In Taiwan, up to 8.9% of people aged ≥15 years have moderate depression [2]. Depression symptoms may include disconsolate emotion, reduced interest in activities, appetite changes, wakefulness or hypersomnia, enervation, attention problems, and suicidal ideation [3,4]. Depression is more prevalent in females than in males [3,5,6]. Only 30% of outpatients with depression receive a clinical diagnosis and treatment [4,6]. Major depressive disorder (MDD) is defined as a severely depressed mood that persists for at least two weeks [6]. The most widely used diagnosis criteria are found in the American Psychiatric Association’s *Diagnostic and Statistical Manual of Mental Disorders, Fourth Edition, Text Revision* (DSM-IV-TR) and the World Health Organization’s *International Classification of Diseases, 11th Revision* (ICD-11), which use the term recurrent depressive disorder [1,6]. Because of the variety of symptoms and the subjective nature of patient assessments, depression diagnosis remains difficult.

The Test of Variables of Attention (TOVA) was developed in the 1960s by Lawrence Greenberg, the Head of the Department of Child and Adolescent Psychiatry, University of Minnesota [6]. It provides healthcare professionals with objective measurements of attention performance and inhibitory control. Visual TOVA aids in the assessment and treatment evaluation of attention deficits, including attention-deficit/hyperactivity disorder, MDD, and other attention-related disorders [7]. Participants’ age, gender, education level, cultural background, and language do not affect its results. TOVA is a continuous performance test commonly used to facilitate the assessment of disease diagnosis and treatment response. Participants are instructed to press the response button when they locate the target figures (squares containing a smaller square near the upper border of the screen) and not to press the button when nontarget figures (squares containing a smaller square near the lower border of the screen) appear. Responses are recorded with a highly precise microswitch (±1 ms). This 21.6-min-long test does not require left–right discrimination or sequencing and is used to calculate response time variability (consistency), response time (speed), commissions (impulsivity), and omissions (focus and vigilance) [8]. These scores assist physicians to diagnose attention deficits.

Quantitative electroencephalography (qEEG) has emerged as a potential intermediate biomarker for the diagnostic clarification of psychological disorders, dysphonetic dyslexia [9], and epileptic seizures [10]. Electroencephalography (EEG) reflects microvolt-level brain electrical activity and is commonly used in neuroscience studies of psychological disorders such as MDD. Noninvasive EEG is safe and requires no surgical procedures; meanwhile, it has a high temporal resolution, which has been used to classify depression in many studies [11] for pharmacologic treatment prediction [11,12,13,14,15,16,17,18] and diagnosis purposes [11,18,19,20,21,22,23]. qEEG is used to evaluate antidepressant treatments, including amitriptyline-changed sleep EEG [24], frontal theta cordance to predict deep brain stimulation [17], frontal theta cordance to predict antidepressant response [16], permutation entropy to predict response to repetitive transcranial magnetic stimulation [14], and increased theta cordance to predict response to ECT [15].

Absolute power is defined as the total energy intensity of an electrode on a certain region at different frequency bands [25]. Relative power is the perceived amount of power of one area or entity in relation to that of another. Researchers [6,26,27] have suggested an association between negative emotion and suppression of affective responses in the bilateral dorsal amygdala and anterior insula. MDD is linked to abnormalities or imbalances in dopamine and serotonin neurotransmitters in the brain, which is associated with stress [28]. EEG electrodes detect abnormalities in the brain’s electrical signals related to such chemical imbalances. The left frontal lobe is involved in brain dysfunction in depression [29]. Knott et al. [30] found that the relative beta power [31] of male individuals with depression was higher than that of healthy controls at 14 electrodes (mainly frontal and occipital areas). Absolute beta power [31] demonstrated significant changes at only six electrodes (frontal area). The use of nonlinear entropy analysis used for the diagnosis of major depression has increased recently. Li [32] reported that, as compared with healthy controls, patients with depression had significantly higher wavelet entropy at 12 electrodes (mainly in the frontal, center, temporal, and occipital areas) in the resting state but not in the mental arithmetic test. Another study [33] noted significant α wave changes in patients with MDD during sleep. EEG signal analysis using frontal brain asymmetry ratio revealed a clear α peak in individuals with depression [34]. According to the above studies, absolute and relative power are common qEEG biomarkers to evaluate the brain activity of MDD patients.

In addition, cordance, a qEEG method, combines complementary information from the absolute and relative power of EEG spectra [35,36]. Cook et al. [31] used absolute and relative power/energy to obtain cordance values. Glucose metabolism rate was indirectly estimated from EEG. Positron emission tomography (PET) operates under the principle that the body’s cells need glucose for energy and that active cells require more energy and therefore absorb more glucose. This method involves labeling glucose with fluorine-18 and injecting it into the blood vessels, and then tracking metabolic changes through imaging [31]. Cook et al. used 35 channels to analyze the correlation between brain metabolic concentration (estimated from PET images) and EEG energy. The relative energy/power of EEG correlates with the PET results, which indicates regional cortical blood flow [37]. A positive correlation was found (*p* < 0.05). Cordance is widely applied for understanding MDD. Cook et al. showed that depressed adults have higher midline cordance than never-depressed subjects. Resting-state qEEG demonstrates differential connectivity in adolescents with MDD, and MDD patients have lower average coherence in the theta band. The pre-treatment frontal qEEG cordance can be used to predict responders to rTMS treatment among MDD patients [38]. However, whether qEEG can differentiate between the brain signals of individuals with different psychiatric disorders remains unclear [39]. Specifically, many researchers [40,41] found that qEEG of theta band cordance relates to MDD patients. Broadway recorded the resting-state EEG from 12 treatment-resistant depression patients at 0, 4, and 24 weeks of deep brain stimulation treatment. Greater frontal theta cordance increases predicted greater decreases in depression severity scores [17]. Early changes in theta cordance of the prefrontal and midline right frontal areas were most predictive of response to antidepressant treatment [41]. An inpatient study of 18 individuals gave similar results to other antidepressant studies, which used prefrontal qEEG (Fp1, Fp2, Fz) cordance to predict the acute outcome of bupropion treatment [40].

Although theta cordance is a well-known qEEG biomarker for the investigation of MDD, whether TOVA affects these classical qEEG biomarkers remains unclear. The aim of this study was to evaluate whether qEEG biomarkers are different with respect to event-related potentials of MDD during target and nontarget trials of TOVA, wherein a trial comprises a 2 s period for each test. We also propose that the scores of approximate entropy from covariance matrix images of EEGs provide the quantization biomarker for separating healthy control and MDD groups and are related to the Beck Depression Inventory (BDI) scores.

## 2. Materials and Methods

This study was performed according to regulation IRB100-121 of Tzu Chi Hospital. The MDD group consisted of patients at the Department of Psychiatry, Tzu Chi General Hospital, Hualien, Taiwan, who had been diagnosed as having MDD (ICD-9: 296.2, 296.3). Subjects with any history of cerebrovascular or cardiovascular disease, schizophrenia, bipolar disorders, anxiety disorders, or epilepsy were excluded. The screening was performed by a physician in a systemic review. We collected data from the MDD group routinely followed by the hospital. The control group (i.e., patients without psychiatric disorders) was matched for gender and age. All subjects were right-handed females in this study. To minimize the influence of pharmacotherapy, the depression medication was temporarily suspended for three days under physician’s supervision before EEG signal collection. Poster advertisements placed throughout the hospital were used to recruit the controls who underwent screening for psychiatric disorders using the Mini-International Neuropsychiatric Interview. The nature of the experiment was explained to all participants, all of whom provided signed informed consent. Both groups completed the Beck Depression Inventory and the Beck Anxiety Inventory. The MDD group also completed the 17-item Hamilton Depression Rating Scale. Table 1 presents the study population demographics.

**Figure 1:**
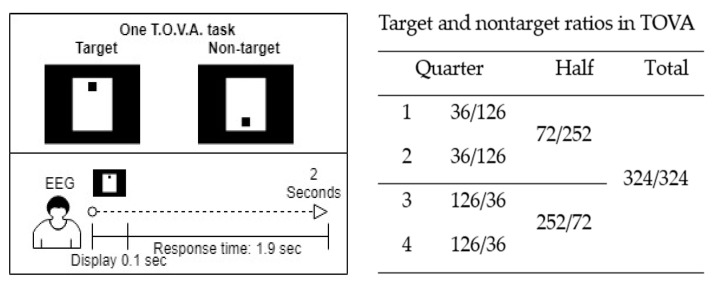
Procedure and target-to-nontarget ratios of the Test of Variables of Attention (TOVA).

The five parameters of TOVA are omission rate, commission rate, mean response time, mean variability, and mean d-prime (response sensitivity) score, which measure the participants’ attention and inhibitory control. The results are divided into four categories: correct responses to targets, correct responses to nontargets, errors of omission (inattention), and errors of omission (impulsivity). Each TOVA trial type is randomly presented every 2 s, comprising 100 ms of display time and 1.9 s of response time. The test consists of 648 stimulus presentations (324 target and 324 nontarget tasks) divided into four quarters with different target and nontarget ratios and has negligible test–retest effects [42]. Figure 1 presents the test procedure.

**Figure 2:**
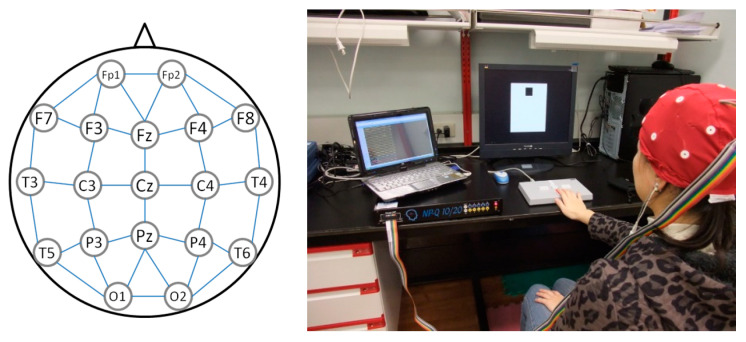
Electrode layout and experimental setup for the Test of Variables of Attention.

All experimental data were collected between 9:00 am and 12:00 pm to eliminate potential diurnal variations. Before undergoing TOVA, all 36 participants filled out clinical surveys, took 2 min of physical rest, and gazed at a black cross at the center of the screen for 5 min as a record of their resting EEG as baseline. MindSet EEG Systems (NP-Q 10/20, NeuroPulse-Systems, LLC, Hawkinsville, GA, USA) were used to record 19 EEG channels, namely Fp1, Fp2, F7, F3, Fz, F4, F8, T3, C3, Cz, C4, T4, T5, P3, Pz, P4, T6, O1, and O2, at a sample rate of 256 Hz. Electrode caps were used to place the recording electrodes over the regions defined by the International 10-20 system, and the ground electrode was placed on an earlobe. Matlab R2019b (Mathworks Inc., Natick, MA, USA) and EEGLAB (Swartz Center for Computational Neuroscience, La Jolla, CA, USA) were used for algorithm development. EEGLAB is an interactive Matlab toolbox for processing continuous and event-related EEG, which allowed us to plot qEEG biomarkers on brain maps and to apply independent component analysis (ICA) for electrooculography (EOG) signal cancelation. Figure 2 depicts the electrode layout and experimental setup.

**Figure 3:**
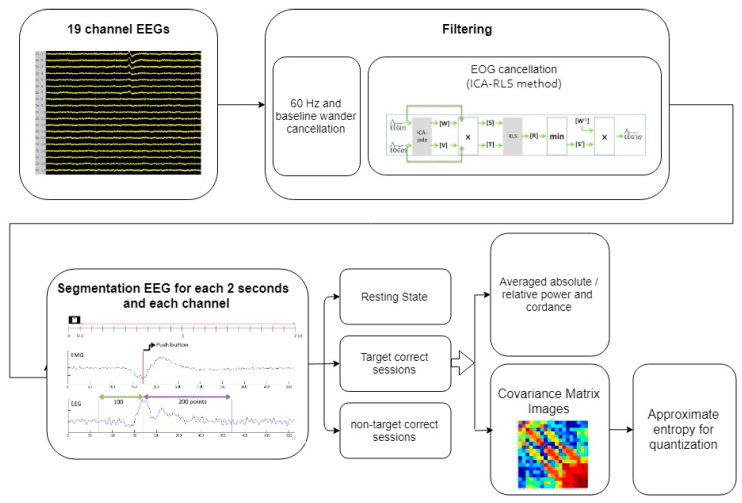
The system block diagram.

In the processing stage, two median filters were used to remove the baseline wander noise, including large-scale (0.2 s) and small-scale (0.04 s) time windows. The 60 Hz artifact was then removed using a finite impulse response band-reject filter. EOG was eliminated using Mosquera’s method [43], which separates the EOG signals from four EOG-related channels (Fp1, Fp2, F7, and F8) by projecting those channels into the ICA space corresponding to other non-EOG channels. The recursive least-squares adaptive filter algorithm was then used to obtain the filtered EEG signals. The filtered EEG signals were partitioned into the 648 stimuli presented every 2 s as signal vector space V. Four responses are possible in TOVA: correct responses to targets ($x_{t}\left(i\right)\in V$), correct responses to nontargets ($x_{\mathrm{nt}\;}\left(i\right)\in V$), omission errors ($\bar{x_{t}}\left(i\right)\in V$), and commission errors ($\bar{x_{\mathrm{nt}\;}}\left(i\right)\in V$), where x represents the signal vector, absolute power, relative power, or cordance. However, for the participants without attention deficits, the numbers of the $\bar{x_{t}}\left(i\right)$ and $\bar{x_{\mathrm{nt}\;}}\left(i\right)$ trials were too small to process. The signal fragments of the correct responses to targets and nontargets were analyzed accordingly. The power calculation was normalized by the total number of the correct responses to targets and nontargets for each person. Hence, the quantitative biomarkers of the EEG channels only focus on target correct, non-target correct, and 5 min resting periods in this study. The averaged absolute power, relative power, and cordance for each person were calculated for comparison and to form covariance matrix images. Finally, the approximate entropy method was applied to quantify the covariance matrix images. The system block diagram is shown in Figure 3.

In Section 2.1, Section 2.2, and Section 2.3, we describe how the biomarkers were generated on brain maps during TOVA. We compare the resting state and the correct responses to targets and nontargets.

### 2.1. Absolute Power, Relative Power, and Cordance

To compute the absolute power of each band $\delta_{\mathrm{abs}}\;$(1–4 Hz), $\theta_{\mathrm{abs}}\;$(4–8 Hz), $\alpha_{\mathrm{abs}}\;$(8–12 Hz), and $\beta_{\mathrm{abs}\;}$(12–16 Hz), the power of the frequency range of each band was added up. The relative power of each band $\delta_{\mathrm{rel}}\;$(1–4 Hz), $\theta_{\mathrm{rel}}\;$(4–8 Hz), $\alpha_{\mathrm{rel}}\;$(8–12 Hz), and $\beta_{\mathrm{rel}\;}$(12–16 Hz) is the percent of the absolute power summed over the four frequency bands. Then, we followed Cook’s method to calculate cordance, which is briefly mentioned as follows.

The absolute and relative power of the channel (c0) and its surroundings (c1–cn) were further averaged to achieve reorganization, which describes the electrode power relative to the surrounding electrodes.

(1) $$\alpha_{{\mathrm{abs}}_{c0}}\;=\;\frac{\left(\alpha_{{\mathrm{abs}}_{c0}}+\sum_{\mathrm{ci}\;=\;1}^{\mathrm{cn}}\alpha_{{\mathrm{abs}}_{\mathrm{ci}}}\right)}{\mathrm{cn}+1}$$

Here, $\alpha_{\mathrm{abs}}$ is the absolute energy. The same method was used to compute the other channels and relative power. As shown in Figure 2, the connections of each channel provide the surrounding signals, which merge as a relative power channel, through this calculation: f3 = (f3 + fp1 + f7 + fz + c3)/5. Values were normalized using z-scores [44] based across channels for each person. The power calculation of each channel was separate, but the frequency bands were calculated together. Absolute power and relative power were also calculated separately. The absolute power $\alpha_{{\mathrm{abs}}_{\mathrm{ch}}}$ and relative power $\alpha_{{\mathrm{rel}}_{\mathrm{ch}}}$ of each channel were summed to obtain the cordance value $\alpha_{{\mathrm{cordance}}_{\mathrm{ch}}}$ as

(2) $$\alpha_{{\mathrm{cordance}}_{\mathrm{ch}}}\;={\;\alpha }_{{\mathrm{abs}}_{\mathrm{ch}}}+\alpha_{{\mathrm{rel}}_{\mathrm{ch}}}$$

where ch(n), n = 1,2...19 is the number of EEG channels.

### 2.2. Covariance Matrix Images

Previously defined $x_{t}\left(i\right)$ and $x_{\mathrm{nt}\;}\left(i\right)$ trials were used to generate covariance matrix images for analysis. The covariance matrix image C(n), a square matrix giving the covariance between each pair of elements of a given random vector, provides information regarding interaction among channels during each TOVA trial. It provides interaction relationships between the 19 channels, and the electrode locations are ordered from frontal to occipital cortex. The covariance matrix image is represented as

(3) $$C(n)=\frac{1}{N}\sum_{i=1}^{n}\frac{X_{i}X_{i}^{T}}{trace\left(X_{i}X_{i}^{T}\right)}$$

where N is the number of correct responses to targets or nontargets. In this study, $X_{i}$ is a vector that represents a time domain EEG signal, including the $x_{t}\left(i\right)$ or $x_{\mathrm{nt}\;}\left(i\right)$ trials. Finally, signal averaging, a signal processing technique [45] was used to increase the signal-to-noise ratio through the calculation $\;\frac{1}{m}\sum_{m}C\left(n\right)$, where m is the number of events of entire $x_{t}\left(i\right)$ or $x_{\mathrm{nt}\;}\left(i\right)$ 2 s trials. That is, a pixel of the covariance matrix image presents a covariance value, which is obtained from two 2 s period EEG signals from two EEG channels during target/nontarget trials. Because the values generated by the covariance matrix are always less than 1, they are normalized and mapped to 8-bit image color between 0 and 255. Finally, on the basis of the probability of covariance values in images, histogram equalization was applied to further enhance the color map.

**Figure 4:**
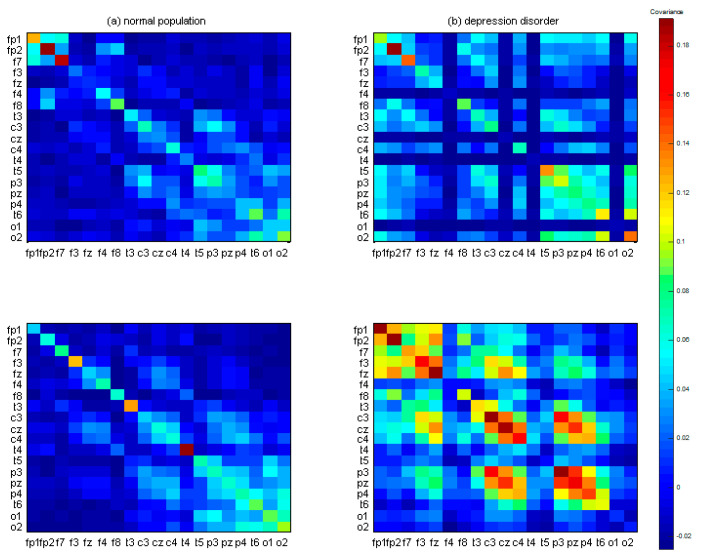
The 19 × 19 covariance matrix images after signal averaging. (**a**) Two randomly selected examples from the control group. (**b**) Two randomly selected examples from the major depressive disorder (MDD) group. Color pixels represent the covariance values.

For instance, $X_{i}$ is a 19 (channels) × 512 (sample points) EEG signal vector used to form the covariance matrix images $X_{i}X_{i}$ of 19 × 19 squares (Figure 4), which constitute the brain pattern images for between-channel interactions. Each color represents different covariance values. The two left-sided figures indicate the covariance matrix images from the normal population with correct target responses; the two right-sided figures indicate the covariance matrix images from the depression group with correct target responses. Some rows or columns in images from the MDD group have more color gaps in adjacent channels than the healthy control group. Therefore, to quantify color gaps in adjacent channels in the covariance matrix images, approximate entropy (ApEn) was used as a potential index to estimate the fluctuations. In Section 2.3, the ApEn method is described in more detail.

### 2.3. Approximate Entropy for Quantization of Covariance Matrix Images

**Figure 5:**
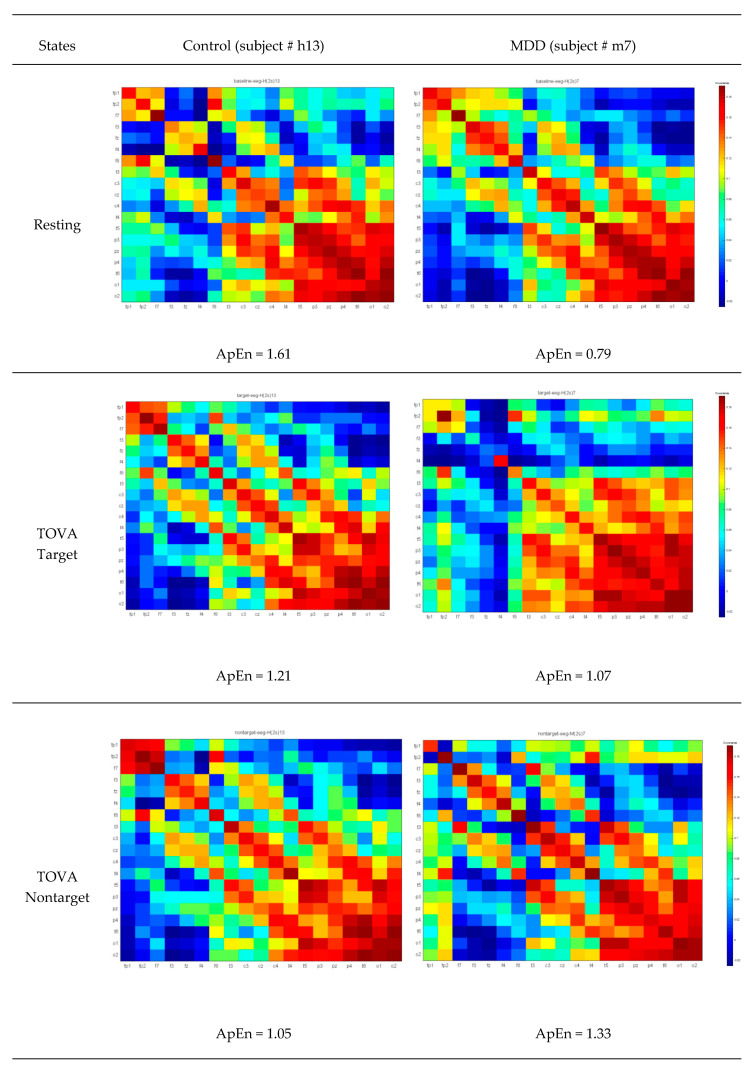
Common covariance matrix images of approximate entropy (ApEn) of participant #h13 in the control group and participant # m7 in the MDD group. Color pixels represent the covariance values.

Approximate entropy, a technique developed by Pincus [46,47], can be used to quantify the predictability of the number sequence. The 19 × 19 covariance matrix image is first reshaped into a single a vector (1 × 361) as a sequence; the reshape order is from left to right and from top to bottom. The ApEn method computes an entropy number from the vector to represent the chaos of the sequence. High ApEn indicates that the signals have low regularity and contain fewer repetitive patterns, whereas low ApEn indicates the presence of numerous repetitive patterns. The ApEn is computed as

(4) $$apen=1n\frac{ApEn(Sn,m1,r)}{ApEn(Sn,m2,r)}$$

where *Sn* is a vector from the reshaped covariance matrix (19 × 19), and the function *ApEn* is defined as $ApEn\left(Sn,\;m,\;r\right)\;=\;mean\left(\frac{N_{imr}}{n-m+1}\right)$.$\;N_{imr}$ indicates the number of counts when the condition |P(i)−P(j)|<r is met, where *P*(*i*) = *Sn*(*i*:*i + m* − 1) and *i* = 1:*n − m* + 1. *r* is the similarity threshold. *m*1 and *m*2 are the lengths of the patterns, and *n* is the length of *Sn*. In the present study, *m*1 and *m*2 were set as 20 and 50. *m*1 equal to 20 is the distance from one diagonal element to the preceding diagonal element. *m*2 equal to 50 is to guarantee the inclusion of the adjacent channel information. Figure 5 displays the common covariance matrix images of one participant in the control and MDD groups, respectively.

### 2.4. Statistical Analysis

Statistical analyses were carried out with the Prism GraphPad Software (version 5, GraphPad Software Inc., San Diego, CA, USA) and MATLAB Software (version R2018b, MathWorks Inc., Natick, MA, USA). Demographic data were analyzed between the control and MDD groups using the Mann–Whitney U test (two-sided, *p* < 0.05). Theta cordance and AnEp values from resting-state, target, and nontarget trials were analyzed between the control and MDD groups using two-way ANOVA with Bonferroni’s multiple comparison test as post hoc to provide the interaction relationships between the two study groups and three TOVA conditions.

In order to provide the analysis performance in the class with fewer ApEn samples, the bootstrapping statistical technique was used to estimate the approximating distribution for original data properties including variance bias, variance, confidence intervals, and prediction error. It is a resampling method to generate a number of resamples, with replacements used to ensure an equal size to the observed dataset in a data distribution sense [48]. Spearman correlation based on ranks using the two-tailed *p* value, which makes no assumption about the distribution of the values, was used to check whether ApEn related to BDI scores. Finally, receiver operating characteristic (ROC) analyses on theta cordance and AnEp scores were used to separately generate area under the curve (AUC) values with exact binomial 95% confidence internal (CI) for each variable, and in combination, to assess measures of prediction accuracy.

## 3. Results

### 3.1. Demographic and Clinical Characteristics

Table 2 presents subject ages, the scores of clinical assessments, and qEEQ biomarkers. There was no difference between the MDD and control groups for age. TOVA scores for the MDD and control groups include correct responses to targets, omission errors (inattention), commission errors (impulsivity), and correct responses to nontargets. The parameters of TOVA are as follows: (1) omission rate—percentage of responses in which targets appear but the response button is not pressed; (2) commission rate—percentage of responses in which nontargets appear but the response button is pressed; (3) mean response time; (4) mean variability; (5) mean d-prime score to analyze the participants’ attention performance and inhibitory control. The results showed significant differences between the two groups and matched the physician’s diagnosis for the MDD patients. The MDD group had higher BDI and BAI scores.

According to Equation (2), cordance is summarized information of absolute and relative power. Therefore, we briefly discuss the results of absolute and relative power in this section, and detailed information is listed in the Supplementary Materials for reference. The essential results including cordance and ApEn are described in the following.

### 3.2. Responses to TOVA

The MDD group had higher error rates (commission rate, *p* < 0.01; omission rate, *p* < 0.05) and had longer reaction times (*p* < 0.01) than the control group. The mean variability (*p* < 0.01) and mean d-prime score (*p* < 0.01) of the MDD group were also significantly different from the control group.

### 3.3. Effects of Groups and Response Conditions on Frontal Theta Cordance

This study focused on theta cordance based on clinical evidence in previous investigations. Table 3 provides the mean and standard deviation of theta cordance. The average theta band energy was lower in the control group than in the MDD group, concentrated in the frontal and occipital lobes (in Table 3); see Supplementary Materials for details.

By applying Bonferroni multiple comparisons as post hoc test, two-way ANOVA was calculated to investigate the effects of different groups (MDD vs. control) and response conditions (resting, target, and nontarget) on frontal theta cordance. Neither group nor response conditions had any effect on frontal theta cordance. Frontal theta cordance of both groups is listed in Table 2.

### 3.4. Effects of Groups and Response Conditions on F7 Theta Cordance

**Figure 6:**
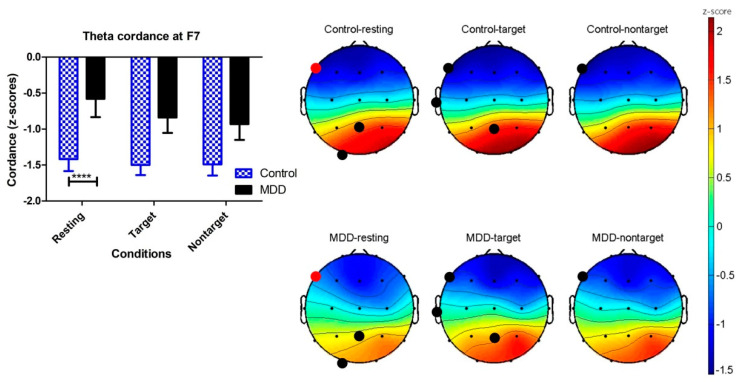
Brain maps of the cordance values (z-scores) in the theta frequency band in the MDD and control groups. Black dots show *p* < 0.05 electroencephalography (EEG) channels by applying unpaired t-test on the control and MDD groups. Red dots are the location of the EEG F7 channels with significant difference using two-way ANOVA (**** *p* < 0.0001).

The F7 theta cordance of both groups is listed in Table 2. Two-way ANOVA was calculated to investigate the effects of the different groups (MDD vs. control) and response conditions (resting, target, and nontarget) on F7 theta cordance. The F7 theta cordance of the control group was lower than the MDD group (*p* < 0.0001). The effect of response conditions was not significant (*p* = 0.5258). The interaction between groups and response conditions did not reach a significant level (*p* = 0.7701). In the resting response condition, Bonferroni multiple comparisons showed that the MDD group had a significantly higher F7 theta cordance than the control group (difference = 0.84, t = 3.029, *p* < 0.01), but not for the target and nontarget conditions. Figure 6 shows the theta cordance brain map in three conditions and the statistical results.

### 3.5. Effects of Groups and Response Conditions on ApEn

**Figure 7:**
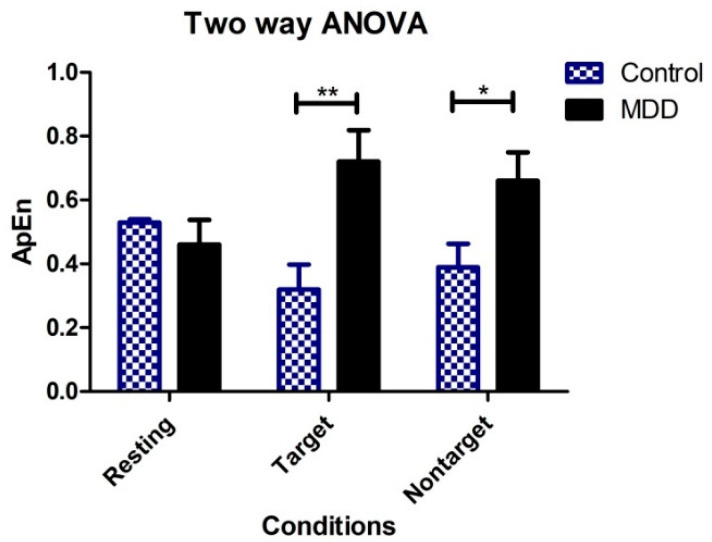
Two-way ANOVA showing group means of ApEn for each of the three conditions (vertical bars represent SD). ** *p* < 0.01, * *p* < 0.05.

Between-group differences in ApEn in the time domain during TOVA were observed, including the control and MDD groups. In Table 2, two-way ANOVA was calculated to investigate the effects of different groups (MDD vs. control) and response conditions (resting, target, and nontarget) on ApEn. There were significant differences in the ApEp between the two groups (*p* < 0.05). The effects of the response conditions were not significant. The interaction between groups and the response conditions was significant (*p* < 0.01). Bonferroni multiple comparisons showed significant differences between the MDD and control groups when the response conditions were target (*p* < 0.01) and nontarget (*p* < 0.05), but not resting, as shown in Figure 7.

### 3.6. Relationship between Severity of Depression and ApEn

**Figure 8:**
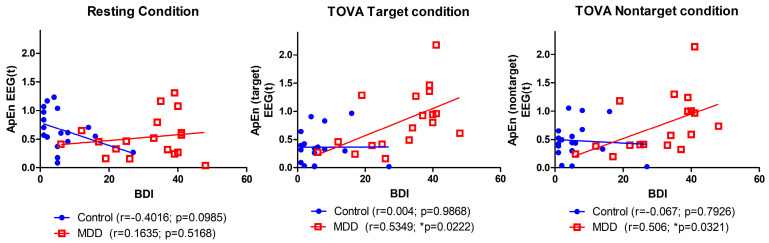
Correlation between BDI score and average ApEn among three conditions.

The correlation between BDI scores and average ApEn for all conditions was found to be moderately positively correlated in the MDD group (r = 0.51, *p* = 0.03). This correlation was not significantly different from zero in the control group (r = −0.19, *p* = 0.43). Figure 8 shows the relationship between BDI and the average ApEn and three different response conditions. The highest correlation (r = 0.535, *p* = 0.02) was for the MDD group and the TOVA target condition, and no significant correlation was found for the control group between any conditions.

### 3.7. Receiver Operating Characteristic Curve

**Figure 9:**
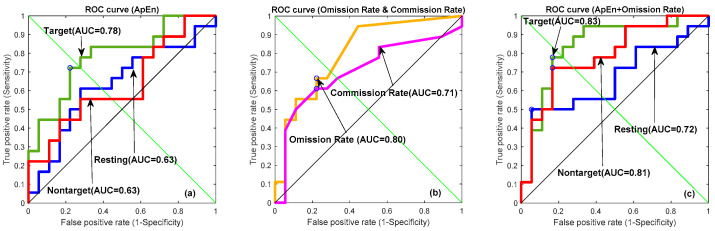
Receiver operating characteristic (ROC) curves depicting different variables, including (**a**) ApEn, (**b**) omission rate (%), and (**c**) a combination of ApEn and 30% omission rate between three conditions (green line: target; red line: nontarget; blue line: resting state; orange line: omission rate (%); and purple line: commission rate (%)).

Receiver operating characteristic (ROC) curve depicted true positive versus false positive rates for the MDD vs. control classification, aiming to assess the discrimination capability of the qEEG variables. ROC curves depict different variables, including (a) ApEn, (b) omission rate (%) and commission rate (%), and (c) a combination of ApEn and omission rate (%), among the three conditions in Figure 9 and Table 4. From Table 4, the omission rate (%) demonstrated a better ROC performance than the commission rate (%). Then, ApEn was combined with different weights of omission rate (%) from 0 to 1 with a 0.1 interval scale. The best performance occurred with the weight equal to 1. In Figure 9a, the target area under the curve (area under the curve, AUC = 0.78) indicated a fair performance for one ApEn variable in differentiating control from depression. At the cut-off point (0.42) suggested by the forward stepwise discriminant function analysis, the sensitivity and specificity for the diagnostic of MDD were 72.22% and 72.22%, respectively. The clinical biomarker, omission rate (%), provides the prediction (AUC = 0.80) in Figure 9b. The performance was further improved in Figure 9c when AnEp was combined with omission rate (%) from the clinical TOVA measures, which was the target ROC curve (AUC = 0.83) with 77.77% accuracy, 83.33% sensitivity, and 72.22% specificity, respectively.

### 3.8. Bootstrapping Simulation

Bootstrapping estimates the properties of an estimator (such as its variance and discriminative ability) by measuring those properties when sampling from a distribution. It derives estimates of standard errors and confidence intervals for complex estimators of the distribution. The method enabled us to observe the shift of ApEn in a confidence interval sense. We computed a sample of 1000 bootstrapped means of random samples taken from the ApEn to make two groups of statistically equal size N = 1000. Bootstrapping techniques were used to internally validate our ApEn model, that is, to simulate the performance with respect to the AUC comparable to our datasets, including target condition (AUC = 0.999, S.E. = 0.0002, 95% C.I. 0.999–1.000), nontarget condition (AUC = 0.983, S.E. = 0.0029, 95% C.I. 0.977–0.989), and resting state (AUC = 0.885, S.E. = 0.0076, 95% C.I. 0.870–0.900).

**Figure 10:**
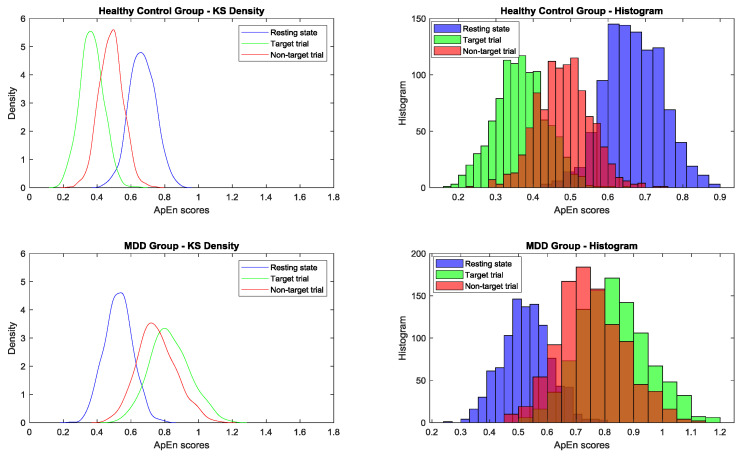
After 1000 bootstrapping simulations for each experimental condition, the kernel smoothing densities and histograms for four groups (from top to bottom: healthy control and MDD for three conditions) (blue: resting state; green: target trial; red: nontarget trial) are plotted.

After 1000 bootstrapping simulations for each experimental condition, the kernel smoothing densities and histograms for four groups with three conditions were plotted in Figure 10. For the healthy group, ApEn density curves and histograms from low to high represented the target trial, nontarget trial, and resting state, respectively; for the MDD group, the orders were reversed. In the resting state, the control group had a larger ApEn than the MDD group. However, during the TOVA test, the MDD group had a greater ApEn in the time domains than the control group. As shown in Figure 5, the ApEn of both groups changed in the testing state, i.e., declining in the control group and increasing in the MDD group.

One-way ANOVA was used to compare the means among 15 conditions between the control and MDD groups to determine mean differences and its 95% confidence intervals of ApEn scores by using bootstrapping simulation. Bonferroni’s multiple comparison test was applied. As shown in Table 5, the 95% confidence interval for the difference between healthy resting state and target condition was positive, and the difference between MDD resting state and target condition was negative. Table 5 and Figure 10 also show the ApEn value shifting among conditions.

The ApEn of the control group in the resting state was higher than that of the MDD group overall. However, during TOVA, this phenomenon was reversed; the ApEn of the control group decreased, and that of all participants with MDD increased. These results indicate that the selected biomarker ApEn is robust between the control and MDD groups. Between-group differences in ApEn were also significant using Bonferroni’s multiple comparison test.

## 4. Discussion

To our knowledge, this is the first study to discriminate MDD patients from healthy patients via the approximate entropy (ApEn) of qEEG when performing an attention task. MDD patients had a higher ApEn than the control group when they performed a cognitive task. The ApEn positively correlated with the severity of depressive symptoms. The ApEn was a better predictor of MDD than frontal theta cordance. Our findings provide evidence for the combination of qEEG and cognitive tasks to discriminate between patients with depression and those without.

We replicated the same attention deficits of MDD patients via TOVA as in our previous study [7]. Major depressive disorder (MDD) is a mental disorder characterized by pervasive low mood and is frequently combined with cognitive deficits [49]. Attention, executive function, and verbal memory are areas of consistent impairment even in remitted MDD patients [50] and mediate poor occupational outcomes [51]. Computerized cognitive tests combined with qEEG are more accessible in terms of cost and in their need for operator training, allowing standardization of assessments, greater consistency and global sensitivity, accuracy in detecting response speed, and they produce reports automatically [52]. As regards the current experimental paradigm, higher rates of omission and commission and longer reaction times in response to a target were robust cognitive dysfunction in MDD patients. Attention and executive function impairments are attributed to neural network abnormalities [49]; therefore, EEG was performed before and during TOVA.

The theta band is closely related to depression and can be used to discriminate between depressed individuals and healthy controls [11,53]. EEG can also be used to predict the response to antidepressant treatments; for example, sleep EEG changes have been used to predict the effect of amitriptyline three weeks later [24]. Cordance derived from qEEG is a quantitative indicator of the nature of brain perfusion [54]. Change in frontal theta cordance can be used to predict response to antidepressants [16,36,40], to deep brain stimulation [17], and to ECT in MDD patients [15]. In an earlier study coupling whole-brain arterial spin labeling perfusion MRI and EEG in healthy adults, theta power was negatively correlated with cortical perfusion in widespread regions [55]. Increased rostral anterior cingulate cortex (rACC) activity is also a promising predictor of treatment response in depression [56], which is negatively correlated to the change of frontal theta cordance [57]. Although an early reduction in frontal theta cordance predicts response to treatments, we demonstrated that theta cordance could not effectively discriminate MDD patients from healthy controls in this study. The small sample size is a possible reason for the nonsignificant result. Another reason is related to the cerebral perfusion characteristic of frontal theta cordance, which is insensitive to changes in easy cognitive tasks. When theta cordance is used to predict treatment response, the perfusion change takes seven days.

It is the first time ApEn has been used as a measure of EEG complexity with MDD patients and healthy controls performing cognitive tasks. The short-term EEG signals of different channels, different response conditions, and groups were quantified as a value and used to discriminate MDD patients and controls. Attention varied significantly in the MDD group; therefore, the ApEn of the target and nontarget conditions was significantly higher. The control group demonstrated no difference from the MDD group in the resting condition. The ApEn positively correlated with the severity of depressive symptoms. This indicates that the signal chaos mode in the MDD group was different from that in the control group; thus, ApEn increased when the MDD group underwent TOVA. By contrast, the ApEn of the control group decreased during TOVA. The bootstrapping simulation provided the positive and negative differences of 95% confidence intervals to verify the ApEn shifting under the influence of TOVA. This finding is similar to that of Li et al. regarding wavelet entropy [32]. As a result of its good sensitivity, ApEn has also been used to process many different physiological signals in studies related to major depressive disorder, including irregular secretion of cortisol and adrenocorticotropin [58,59,60,61], diurnal elevations in plasma interleukin-6 levels [62], gastric dysrhythmia [63], cardiorespiratory coupling [64], heart rate variability [65,66], and EEG signals [22].

Average ApEn (in resting, target, and nontarget conditions) was positively correlated with the severity of depression in the MDD group, but not the control group. The significant association between ApEn and BDI score reveals the methods have excellent validity as a biomarker of major depression. ApEn was closely related to symptoms of depression. It is reasonable to propose that ApEn will be a good predictor for treatment response in depression. Permutation entropy derived from qEEG predicts the effect of repetitive transcranial magnetic stimulation [14]. Our findings provide evidence for the combination of qEEG and cognitive tasks to discriminate between patients with depression and those without.

## 5. Conclusions

The integration of qEEG and TOVA can successfully distinguish the differences in attention deficits between participants with MDD and controls. The ApEn of the control group decreased and the ApEn of the MDD group increased during TOVA. Specifically, during the attention test, the level of chaos increased for the MDD group and decreased for the control group. This method can be applied in the diagnosis of cognitive function and the evaluation of the degree of recovery in individuals with MDD.

## Figures and Tables

**Table 1:** Study population demographics.

	Control (*n* = 18)	MDD (*n* = 18)	*p* Values
Age	40.7 ± 15.8	42.8 ± 10.4	
HAM-D-17	x	20.4 ± 8.47	
BAI	3.33 ± 2.61	27.7 ± 9.87	*** (*p* < 0.0001)
BDI	6.22 ± 6..76	30.8 ± 11.7	*** (*p* < 0.0001)

*** *p* < 0.0001. x represents no HAM-D-17 score on control group. HAM-D-17: 17-item Hamilton Depression Rating Scale; BAI: Beck Anxiety Inventory; BDI: Beck Depression Inventory.

**Table 2:** Demographic data, TOVA, theta cordance, and ApEn of the MDD and control groups.

Variables	Control (*n* = 18)	MDD (*n* = 18)	*p* Values
Age	40.7 ± 15.8	42.8 ± 10.4	*p* = 0.7274
HAM-D-17	NA	20.4 ± 8.47	
BAI	3.33 ± 2.61	27.7 ± 9.87	*p* < 0.0001
BDI	6.22 ± 6.76	30.8 ± 11.7	*p* < 0.0001
**TOVA**			
Omission Rate (%)	1.70 ± 5.55	7.29 ± 13.0	*p* = 0.0016
Commission Rate (%)	2.06 ± 2.18	3.33 ± 2.36	*p* = 0.0358
Mean Response Time (ms)	363 ± 47.8	450 ± 92.5	*p* = 0.0012
Mean Variability (ms)	75.7 ± 29.9	128 ± 52.6	*p* = 0.0009
Mean d-prime score (ms)	5.50 ± 1.25	3.98 ± 1.2	*p* = 0.0007
**Frontal Theta Cordance**			
Resting	−1.37 ± 0.82	−1.03 ± 1.04	NS
Target	−1.34 ± 0.85	−1.20 ± 0.73	NS
Nontarget	−1.41 ± 0.78	−1.30 ± 0.73	NS
**F7 Theta Cordance**			
Resting	−1.42 ± 0.70	−0.58 ± 1.08	*p* < 0.01
Target	−1.50 ± 0.59	−0.84 ± 0.91	NS
Nontarget	−1.49 ± 0.66	−0.93 ± 0.94	NS
**ApEn**			
Resting	0.53 ± 0.40	0.46 ± 0.33	NS
Target	0.32 ± 0.33	0.72 ± 0.42	*p* < 0.01
Nontarget	0.39 ± 0.31	0.66 ± 0.38	*p* < 0.05

HAM-D−17: 17-item Hamilton Depression Rating Scale; BAI: Beck Anxiety Inventory; BDI: Beck Depression Inventory; NA: not available; NS: not significant.

**Table 3:** T-test results of differences between the MDD and control groups regarding cordance in the theta band channels. (z-score).

	Resting			Target			Nontarget		
	Control	Depression	*p*	Control	Depression	*p*	Control	Depression	*p*
Fp1	−1.48 ± 0.84	−0.98 ± 1.11	0.135	−1.44 ± 0.81	−1.13 ± 0.84	0.26	−1.51 ± 0.78	−1.19 ± 0.87	0.25
Fp2	−1.38 ± 0.76	−0.99 ± 1.07	0.22	−1.40 ± 0.83	−1.26 ± 0.65	0.571	−1.47 ± 0.73	−1.42 ± 0.60	0.805
F7	−1.42 ± 0.70	−0.58 ± 1.08	**0.009**	−1.50 ± 0.59	−0.84 ± 0.91	**0.014**	−1.49 ± 0.66	−0.93 ± 0.94	**0.048**
F3	−1.16 ± 0.66	−0.80 ± 0.81	0.147	−1.18 ± 0.65	−0.82 ± 0.93	0.189	−1.18 ± 0.69	−0.85 ± 0.91	0.222
Fz	−1.26 ± 0.85	−1.11 ± 0.94	0.619	−1.19 ± 0.91	−1.21 ± 0.71	0.941	−1.26 ± 0.84	−1.28 ± 0.73	0.936
F4	−0.99 ± 0.51	−0.85 ± 0.73	0.508	−1.00 ± 0.68	−0.91 ± 0.46	0.633	−1.06 ± 0.57	−1.03 ± 0.40	0.837
F8	−1.13 ± 0.56	−0.72 ± 1.05	0.146	−1.32 ± 0.70	−1.12 ± 0.85	0.437	−1.40 ± 0.62	−1.28 ± 0.93	0.649

Bold font denotes significant after post hoc test.

**Table 4:** Parameters of ROC curves of ApEn, omission rate (%), commission rate (%), and a combination of ApEn and omission rate.

Receiver Operating Characteristic (ROC) Curve	Area Under the Curve (AUC)	S.E.	95% C.I.	Performance
**ApEn**				
Resting	0.63	0.094	(0.45, 0.81)	Poor
Target	0.78	0.078	(0.63, 0.94)	Fair
Nontarget	0.63	0.093	(0.45, 0.82)	Poor
**Omission Rate (%)**				
TOVA Scores	0.80	0.074	(0.66, 0.95)	Good
**Commission Rate (%)**				
TOVA Scores	0.71	0.087	(0.53, 0.88)	Good
**ApEn+ Omission Rate** **(%)**				
Resting	0.72	0.086	(0.55, 0.89)	Fair
Target	0.83	0.070	(0.70, 0.96)	Good
Nontarget	0.81	0.072	(0.67, 0.96)	Good

AUC: Area under the curve; S.E.: standard error; 95% C.I.: 95% confidence interval.

**Table 5:** One-way ANOVA results of mean differences and its 95% confidence intervals in the approximate entropy among conditions.

Bonferroni’s Multiple Comparison Test	Mean Diff.	95% CI of Diff
H_resting vs. H_Target	0.2950	0.2767 to 0.3134
H_resting vs. H_nontarget	0.1824	0.1640 to 0.2007
H_resting vs. M_resting	0.1356	0.1172 to 0.1539
H_resting vs. M_Target	−0.1623	−0.1806 to −0.1439
H_resting vs. M_nontarget	−0.0849	−0.1033 to −0.0666
H_Target vs. H_nontarget	−0.1126	−0.1310 to −0.0943
H_Target vs. M_resting	−0.1594	−0.1778 to −0.1411
H_Target vs. M_Target	−0.4573	−0.4756 to −0.4389
H_Target vs. M_nontarget	−0.3799	−0.3983 to −0.3616
H_nontarget vs. M_resting	−0.0468	−0.06514 to −0.0285
H_nontarget vs. M_Target	−0.3446	−0.3630 to −0.3263
H_nontarget vs. M_nontarget	−0.2673	−0.2856 to −0.2489
M_resting vs. M_Target	−0.2978	−0.3162 to −0.2795
M_resting vs. M_nontarget	−0.2205	−0.2388 to −0.2022
M_Target vs. M_nontarget	0.0774	0.0590 to 0.0957

## Supplementary Materials

The following are available online at https://www.mdpi.com/2076-3425/10/11/828/s1, Figure S1: Brain maps of average values of absolute energy in the (a) delta, (b) theta, (c) alpha, and (d) beta frequency bands in the MDD and control groups, Figure S2: Brain maps of the average values of the relative energy in the (a) delta, (b) theta, (c) alpha, and (d) beta frequency bands in the MDD and control groups, Figure S3: Brain maps of the cordance values of the relative energy in the delta (a), theta (b), alpha (c), and beta (d) frequency bands in the MDD and control groups, Table S1: Four band absolute power (unpaired t test), Table S2: Four band relative power (unpaired t test), Table S3: Four band cordance (unpaired t test).
