# Supplementary material for: The Changes of qEEG Approximate Entropy during Test of Variables of Attention as a Predictor of Major Depressive Disorder
Source: Brain Sci. 2020 Nov 7;10(11):828. doi: 10.3390/brainsci10110828 (PMC7695214; doi:10.3390/brainsci10110828)
Supplement: Supplementary file 1 [file brainsci-10-00828-s001.pdf]

Article

The Changes of qEEG Approximate Entropy during Test of Variables of Attention as a Predictor of Major Depressive Disorder

Supplementary material

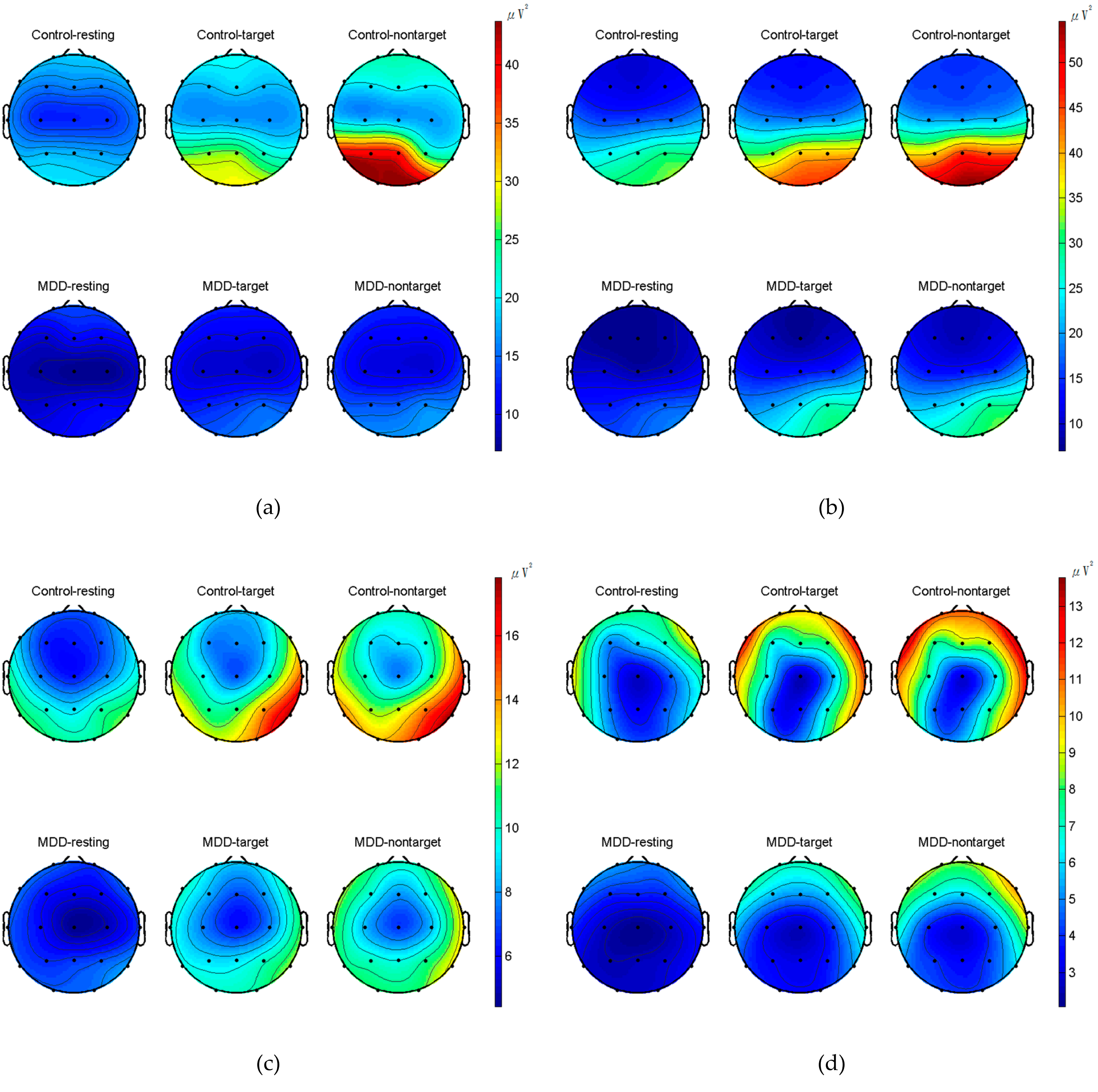

Figure S1. Brain maps of average values of absolute energy in the (**a**) delta, (**b**) theta, (**c**) alpha, and (**d**) beta frequency bands in the MDD and control groups.

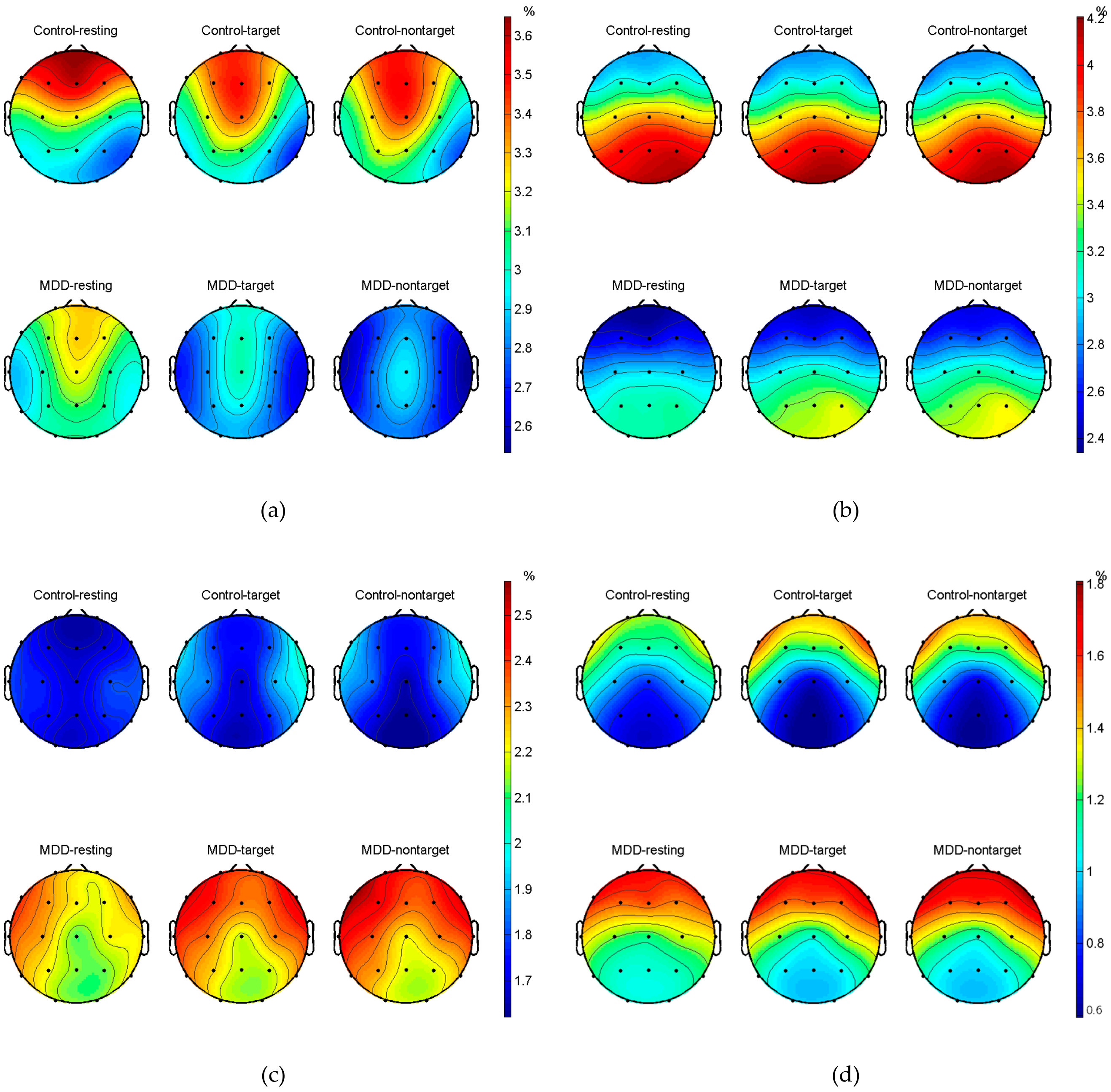

Figure S2. Brain maps of the average values of the relative energy in the (**a**) delta, (**b**) theta, (**c**) alpha, and (**d**) beta frequency bands in the MDD and control groups.

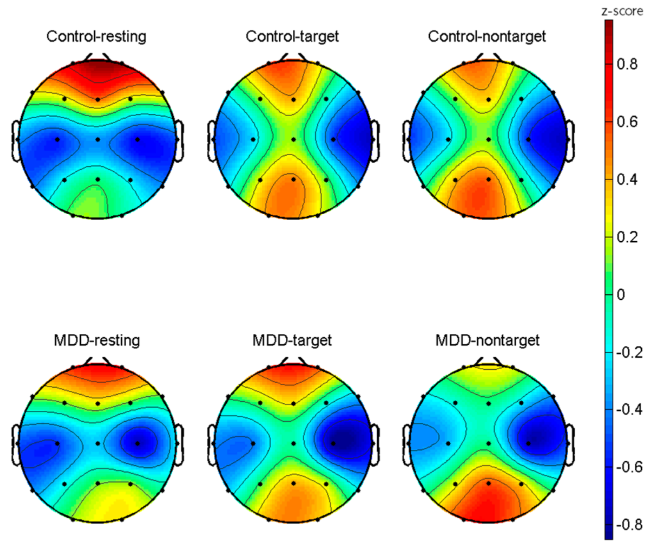

(a)

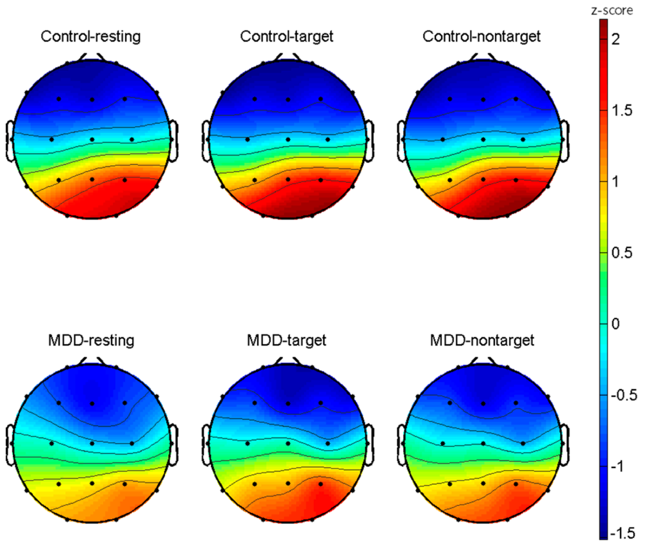

(b)–

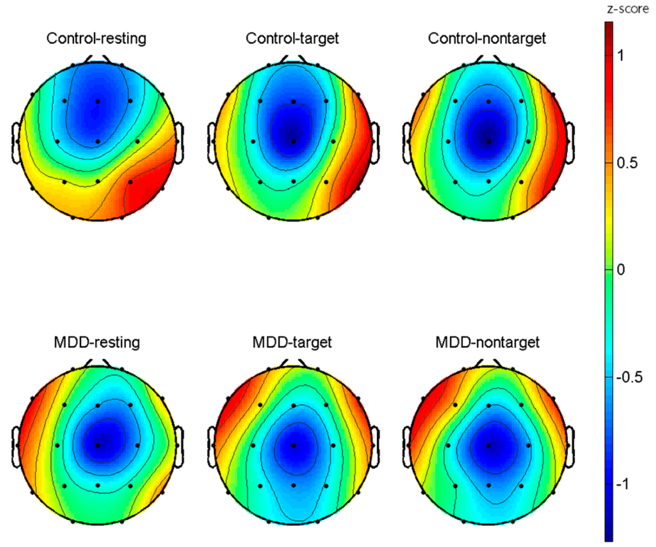

(c)

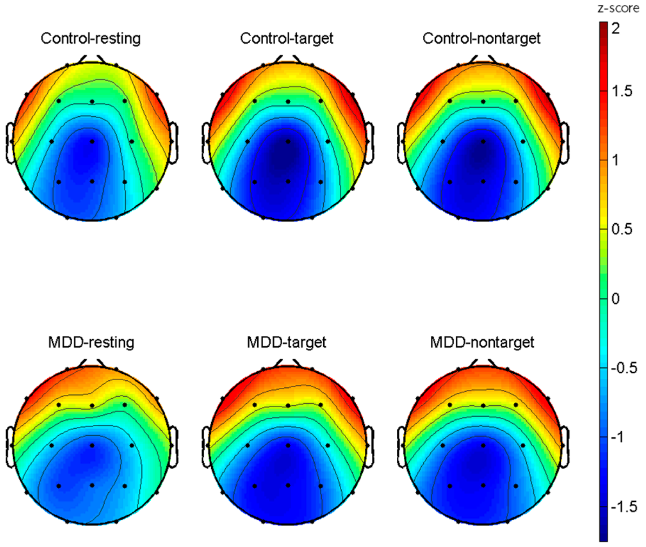

(d)–

Figure S3. Brain maps of the cordance values of the relative energy in the delta (**a**), theta (**b**), alpha (**c**), and beta (**d**) frequency bands in the MDD and control groups.

Table S1. Four band absolute power (unpaired t test).

**delta**	**Control**	**MDD**	**t**	***p***	**theta**					**alpha**					**beta**	
baseline	18.48±10.33	13.68±12.28	1.269	0.213	11.01±5.55	7.98±8.01	1.319	0.196	7.31±5.12	7.37±5.12	-0.036	0.972	7.97±11.08	5.26±3.08	1	0.324
	18.90±9.79	13.68±12.77	1.376	0.178	11.22±4.95	8.00±7.69	1.498	0.143	7.32±4.91	6.73±4.51	0.375	0.71	7.80±11.07	4.83±2.89	1.102	0.278
	17.35±9.28	11.04±5.65	2.466	0.019	12.25±5.83	8.07±4.95	2.317	0.027	8.60±7.11	7.50±3.82	0.578	0.567	8.64±12.36	5.01±2.60	1.22	0.231
	15.56±7.23	9.48±4.46	3.041	0.005	11.61±5.22	7.03±4.05	2.943	0.006	6.43±3.60	6.17±3.10	0.231	0.819	5.07±4.11	3.99±2.01	0.999	0.325
	16.11±8.11	11.59±10.29	1.463	0.153	10.94±5.50	7.19±6.63	1.848	0.073	6.38±4.07	5.75±3.92	0.475	0.638	6.02±8.28	3.91±2.24	1.046	0.303
	15.85±7.08	11.00±11.36	1.538	0.133	12.44±5.05	8.19±8.44	1.836	0.075	7.00±4.58	5.92±3.98	0.757	0.454	6.91±10.37	3.99±2.51	1.161	0.254
	17.86±9.21	12.85±14.31	1.25	0.22	13.11±5.86	9.49±10.46	1.278	0.21	8.75±8.46	7.42±5.38	0.561	0.578	9.80±17.94	5.22±3.45	1.063	0.295
	16.48±9.13	8.63±3.55	3.399	0.002	18.31±9.22	10.40±9.15	2.583	0.014	10.82±12.85	7.23±2.89	1.156	0.256	9.58±18.63	4.03±1.67	1.259	0.217
	12.60±4.99	7.89±4.51	2.971	0.005	15.51±7.46	10.33±11.56	1.599	0.119	7.57±5.72	6.11±3.90	0.894	0.377	5.12±8.33	2.72±0.89	1.217	0.232
	12.56±5.45	6.91±2.85	3.895	0	16.04±8.73	8.49±8.71	2.597	0.014	6.29±2.99	4.42±2.09	2.17	0.037	2.77±1.53	2.07±0.71	1.766	0.086
	12.96±5.16	6.88±3.72	4.061	0	17.30±8.85	9.57±12.22	2.173	0.037	7.80±4.84	5.04±2.83	2.088	0.044	4.55±4.76	2.69±1.48	1.58	0.123
	15.88±7.67	9.49±5.32	2.905	0.006	20.61±12.26	12.16±14.37	1.897	0.066	10.02±8.83	6.74±3.58	1.459	0.154	7.45±10.62	3.92±2.25	1.377	0.177
	18.26±13.38	9.62±6.62	2.458	0.019	24.69±12.49	14.41±18.18	1.977	0.056	11.36±12.86	7.14±3.87	1.335	0.191	7.86±17.75	2.91±0.99	1.181	0.246
	18.04±10.63	9.46±6.05	2.975	0.005	24.82±12.23	13.98±16.80	2.214	0.034	9.83±6.67	6.57±3.70	1.812	0.079	4.79±6.47	2.53±0.89	1.474	0.15
	17.65±7.47	10.42±7.75	2.849	0.007	27.12±13.96	15.44±19.54	2.064	0.047	8.98±3.61	6.76±4.52	1.627	0.113	3.34±1.52	2.61±1.12	1.646	0.109
	17.18±8.05	11.12±8.21	2.237	0.032	29.97±18.51	16.73±20.47	2.036	0.05	10.48±5.52	7.12±5.24	1.87	0.07	4.45±3.54	2.86±1.24	1.799	0.081
	17.34±8.29	11.60±9.28	1.958	0.058	30.94±18.58	17.82±22.72	1.896	0.066	11.62±8.79	8.07±5.54	1.447	0.157	6.28±9.30	3.53±1.64	1.232	0.227
	19.84±10.99	10.84±7.77	2.837	0.008	30.33±15.49	16.15±19.28	2.434	0.02	10.81±6.78	7.21±4.32	1.901	0.066	4.98±6.51	2.76±1.23	1.424	0.163
	19.39±9.00	12.02±9.45	2.395	0.022	32.11±17.77	18.02±23.08	2.053	0.048	10.62±4.86	7.50±5.69	1.774	0.085	4.45±3.57	2.91±1.37	1.704	0.098
target	23.40±15.01	12.92±7.83	2.625	0.013	15.50±9.93	9.72±4.97	2.206	0.034	10.55±4.47	10.72±6.97	-0.089	0.93	11.52±10.95	8.57±8.35	0.911	0.369
	23.14±13.69	12.71±8.43	2.753	0.009	15.46±9.63	9.63±5.73	2.209	0.034	10.59±4.67	10.46±6.59	0.069	0.945	11.45±10.76	8.98±8.13	0.775	0.444
	21.01±11.98	13.15±7.31	2.376	0.023	16.28±9.46	12.08±8.00	1.436	0.16	12.36±7.39	11.85±6.82	0.218	0.829	13.79±18.76	8.71±7.50	1.067	0.294
	19.70±11.05	11.23±5.79	2.879	0.007	15.97±10.73	10.19±5.81	2.008	0.053	8.99±3.56	9.75±5.16	-0.519	0.607	7.66±6.15	6.80±5.75	0.436	0.666
	21.02±12.45	11.09±7.04	2.946	0.006	15.63±11.24	8.77±5.28	2.345	0.025	9.08±3.87	8.01±4.51	0.761	0.452	8.74±7.68	6.01±4.97	1.266	0.214
	20.09±9.75	10.94±6.14	3.37	0.002	16.58±10.10	10.53±7.63	2.027	0.051	9.83±4.44	9.45±4.85	0.244	0.809	9.04±8.69	7.47±6.17	0.622	0.538
	21.77±10.89	12.16±8.34	2.969	0.005	16.83±8.40	11.91±9.64	1.634	0.111	13.50±7.81	12.57±9.02	0.33	0.743	13.71±16.30	10.59±9.74	0.699	0.49
	19.76±11.34	13.20±12.28	1.664	0.105	23.56±15.93	17.06±16.18	1.215	0.233	12.73±8.07	11.86±6.31	0.36	0.721	11.35±19.44	7.31±4.91	0.855	0.399
	17.60±10.09	10.49±7.21	2.432	0.02	20.37±12.33	13.42±12.59	1.672	0.104	10.21±6.58	8.43±4.10	0.977	0.335	7.19±13.51	3.85±1.56	1.041	0.305
	17.41±10.60	10.04±7.04	2.456	0.019	20.48±13.66	13.31±15.58	1.467	0.152	7.67±4.14	6.77±3.78	0.681	0.5	3.01±1.36	2.97±1.17	0.091	0.928
	17.06±9.49	9.90±7.26	2.542	0.016	21.64±12.94	14.95±18.85	1.241	0.223	10.61±5.41	8.51±5.49	1.158	0.255	6.19±6.07	4.84±3.82	0.8	0.429

	19.94±9.84	13.74±12.11	1.687	0.101	26.08±15.06	21.24±25.05	0.702	0.488	15.86±15.78	13.25±8.31	0.621	0.538	12.75±22.72	9.02±7.24	0.663	0.512
	43.75±74.57	14.76±15.34	1.616	0.115	40.49±34.06	22.45±26.05	1.785	0.083	14.34±9.34	10.97±6.90	1.234	0.226	9.42±18.79	4.81±2.49	1.032	0.309
	39.88±59.29	14.29±13.79	1.783	0.083	38.66±30.54	21.83±26.37	1.769	0.086	11.79±5.17	10.15±6.74	0.822	0.417	4.49±2.48	4.01±1.93	0.646	0.523
	38.48±49.30	14.29±13.00	2.013	0.052	45.69±40.24	24.06±33.18	1.759	0.088	12.11±5.59	9.62±7.47	1.133	0.265	4.14±1.87	3.48±1.49	1.182	0.245
	24.26±16.76	16.11±17.17	1.441	0.159	44.23±37.69	28.84±40.99	1.172	0.249	14.50±10.92	10.76±9.00	1.12	0.27	7.38±12.66	4.24±1.86	1.044	0.304
	24.49±17.64	16.41±17.15	1.393	0.173	47.23±41.12	30.61±41.61	1.206	0.236	17.82±16.04	12.84±9.52	1.131	0.266	11.24±22.28	6.12±3.79	0.962	0.343
	43.33±59.95	16.40±16.56	1.837	0.075	52.04±47.12	27.04±34.13	1.823	0.077	13.46±6.21	11.12±8.36	0.954	0.347	4.92±2.77	4.27±2.24	0.783	0.439
	43.23±59.35	16.91±17.80	1.802	0.08	54.62±49.54	31.13±45.76	1.478	0.149	15.75±10.78	11.26±10.27	1.281	0.209	7.73±12.73	4.23±2.12	1.153	0.257
nontarget	20.91±15.05	12.50±6.08	2.199	0.035	13.47±9.79	8.37±3.86	2.053	0.048	8.92±4.10	9.14±4.31	-0.158	0.876	10.16±11.03	7.23±5.39	1.011	0.319
	20.29±14.55	12.34±6.30	2.128	0.041	13.29±9.49	8.24±4.63	2.028	0.05	8.87±4.26	8.92±4.35	-0.032	0.975	10.02±10.76	7.47±5.73	0.887	0.381
	18.79±12.43	12.40±5.25	2.009	0.053	14.57±8.96	10.57±6.61	1.522	0.137	10.91±7.12	10.23±3.66	0.359	0.722	12.43±17.45	7.46±4.31	1.175	0.248
	18.06±12.65	10.61±4.24	2.37	0.024	14.22±10.51	9.06±5.18	1.867	0.071	7.76±3.19	8.33±3.02	-0.553	0.584	6.62±5.95	5.77±3.32	0.53	0.6
	19.27±13.60	10.60±5.26	2.525	0.016	13.66±10.88	7.63±4.66	2.16	0.038	7.79±3.43	7.01±3.33	0.699	0.49	7.58±7.49	5.28±3.97	1.151	0.258
	18.05±12.01	10.29±4.68	2.556	0.015	14.54±9.68	9.30±7.50	1.815	0.078	8.50±4.11	8.02±3.12	0.39	0.699	8.15±9.01	6.00±4.23	0.916	0.366
	18.99±11.95	11.27±6.00	2.449	0.02	14.79±8.24	10.15±8.48	1.662	0.106	12.01±8.05	10.58±6.03	0.601	0.552	12.51±16.77	8.55±6.48	0.935	0.356
	18.19±10.88	11.24±7.00	2.28	0.029	21.62±13.45	14.57±13.00	1.6	0.119	11.91±7.84	9.94±4.15	0.941	0.353	10.71±17.89	5.83±2.62	1.145	0.26
	16.83±10.67	9.41±5.61	2.612	0.013	18.89±11.35	12.55±12.44	1.595	0.12	9.55±6.40	7.66±4.18	1.052	0.3	6.63±12.39	3.47±1.11	1.078	0.289
	16.72±11.16	9.50±5.84	2.433	0.02	18.36±12.02	12.96±16.84	1.108	0.276	7.14±4.02	6.21±3.71	0.72	0.477	2.64±1.17	2.64±0.78	-0.017	0.987
	16.37±10.20	8.94±6.07	2.655	0.012	19.57±11.24	14.29±20.77	0.949	0.349	10.10±5.53	7.70±4.76	1.395	0.172	5.65±6.05	4.20±2.87	0.917	0.366
	17.89±10.58	11.97±9.24	1.789	0.083	23.45±13.62	19.27±25.54	0.613	0.544	15.22±17.24	11.49±6.95	0.852	0.4	11.86±22.26	7.29±5.36	0.847	0.403
	27.33±31.63	12.56±10.91	1.874	0.07	33.88±26.24	19.79±23.27	1.704	0.098	13.17±8.20	9.77±5.78	1.441	0.159	8.74±17.16	4.12±1.82	1.135	0.264
	26.63±26.15	12.62±9.88	2.126	0.041	32.49±24.51	20.08±25.27	1.496	0.144	10.74±4.63	9.04±5.85	0.969	0.339	3.95±2.13	3.42±1.35	0.893	0.378
	26.81±22.93	13.44±11.52	2.209	0.034	38.49±36.22	22.94±33.62	1.335	0.191	11.02±4.99	9.05±7.17	0.96	0.344	3.58±1.52	3.15±1.29	0.908	0.37
	22.48±16.63	14.76±13.77	1.517	0.139	39.20±34.78	27.51±42.45	0.904	0.372	14.04±11.81	10.10±8.69	1.14	0.262	6.82±12.16	3.71±1.54	1.078	0.288
	22.35±17.24	14.49±14.06	1.498	0.143	42.08±38.66	28.36±42.01	1.02	0.315	17.48±17.46	11.84±9.30	1.209	0.235	10.50±21.75	5.29±3.34	1.006	0.322
	29.39±27.90	14.53±12.55	2.061	0.047	43.97±42.03	24.47±31.95	1.566	0.127	12.33±5.59	10.02±7.35	1.06	0.297	4.36±2.45	3.64±1.66	1.028	0.311
	29.10±27.38	15.55±15.27	1.834	0.075	45.75±45.23	29.28±46.20	1.08	0.288	14.75±11.58	10.54±9.68	1.184	0.245	7.01±12.21	3.71±1.79	1.137	0.263

Table S2. Four band relative power (unpaired t test).

**delta**	**Control**	**MDD**	**t**	***p***	**theta**				alpha				beta			
baseline	3.65±0.87	3.23±0.88	1.44	0.159	2.87±0.66	2.34±0.63	2.435	0.02	1.67±0.47	2.30±0.55	-3.694	0.001	1.30±0.70	1.67±0.52	-1.782	0.084
	3.65±0.87	3.30±0.94	1.164	0.253	2.89±0.65	2.38±0.68	2.316	0.027	1.67±0.45	2.23±0.53	-3.39	0.002	1.26±0.69	1.63±0.52	-1.801	0.081
	3.45±0.87	3.01±0.78	1.596	0.12	2.90±0.73	2.46±0.77	1.767	0.086	1.78±0.45	2.41±0.59	-3.589	0.001	1.38±0.72	1.65±0.55	-1.302	0.202
	3.50±0.83	3.15±0.83	1.276	0.211	3.11±0.67	2.53±0.78	2.383	0.023	1.70±0.40	2.31±0.60	-3.589	0.001	1.15±0.53	1.53±0.43	-2.366	0.024
	3.60±0.83	3.28±0.86	1.13	0.267	3.05±0.64	2.47±0.69	2.622	0.013	1.66±0.42	2.22±0.55	-3.453	0.002	1.14±0.51	1.56±0.42	-2.657	0.012
	3.44±0.84	3.22±0.88	0.744	0.462	3.17±0.64	2.60±0.82	2.308	0.027	1.69±0.35	2.20±0.52	-3.461	0.001	1.16±0.56	1.50±0.45	-2.017	0.052
	3.39±0.87	3.10±0.81	1.039	0.306	2.98±0.68	2.51±0.80	1.876	0.069	1.75±0.37	2.32±0.53	-3.703	0.001	1.37±0.75	1.60±0.53	-1.101	0.279
	3.10±0.92	2.88±0.78	0.787	0.437	3.39±0.92	2.84±1.10	1.623	0.114	1.80±0.45	2.38±0.73	-2.861	0.007	1.20±0.67	1.41±0.55	-1.069	0.292
	3.17±0.88	3.01±0.87	0.549	0.587	3.54±0.82	2.94±1.06	1.913	0.064	1.76±0.44	2.29±0.78	-2.535	0.016	0.97±0.41	1.25±0.40	-2.092	0.044
	3.22±0.89	3.23±0.95	-0.02	0.985	3.70±0.84	2.97±1.05	2.321	0.026	1.69±0.45	2.12±0.70	-2.143	0.039	0.81±0.38	1.19±0.38	-3.007	0.005
	3.06±0.85	3.05±0.81	0.011	0.992	3.61±0.78	2.93±1.07	2.177	0.036	1.80±0.43	2.22±0.65	-2.295	0.028	0.99±0.36	1.31±0.42	-2.446	0.02
	2.98±0.91	2.99±0.79	-0.04	0.968	3.53±0.84	2.90±1.06	1.966	0.058	1.82±0.43	2.24±0.56	-2.499	0.017	1.15±0.53	1.38±0.49	-1.389	0.174
	2.94±1.05	2.93±0.87	0.028	0.978	3.86±0.95	3.09±1.17	2.172	0.037	1.77±0.53	2.30±0.81	-2.318	0.027	0.89±0.48	1.18±0.52	-1.783	0.084
	3.00±1.01	3.04±0.94	-0.127	0.9	3.96±0.96	3.16±1.23	2.173	0.037	1.74±0.56	2.21±0.83	-1.97	0.057	0.75±0.32	1.09±0.44	-2.664	0.012
	2.99±1.00	3.11±0.92	-0.394	0.696	4.06±0.95	3.15±1.12	2.62	0.013	1.69±0.54	2.14±0.78	-1.98	0.056	0.70±0.28	1.09±0.44	-3.108	0.004
	2.83±0.99	3.05±0.96	-0.692	0.494	4.09±1.00	3.20±1.21	2.391	0.022	1.77±0.54	2.13±0.74	-1.649	0.108	0.77±0.33	1.11±0.51	-2.412	0.021
	2.75±0.99	2.95±0.87	-0.629	0.534	4.04±0.96	3.12±1.14	2.619	0.013	1.79±0.50	2.23±0.70	-2.193	0.035	0.89±0.40	1.20±0.53	-1.991	0.055
	2.93±1.04	3.05±0.91	-0.368	0.715	4.12±0.96	3.18±1.17	2.64	0.012	1.69±0.54	2.17±0.80	-2.1	0.043	0.70±0.28	1.09±0.48	-3.011	0.005
	2.83±1.04	3.05±0.95	-0.65	0.52	4.18±0.99	3.20±1.17	2.694	0.011	1.72±0.55	2.14±0.76	-1.9	0.066	0.72±0.28	1.10±0.53	-2.695	0.011
target	3.49±1.03	2.78±0.81	2.27	0.03	2.80±0.87	2.52±0.66	1.09	0.283	1.79±0.58	2.49±0.63	-3.454	0.001	1.44±0.73	1.73±0.72	-1.196	0.24
	3.41±0.97	2.82±0.77	2.015	0.052	2.83±0.86	2.53±0.69	1.165	0.252	1.81±0.53	2.43±0.60	-3.27	0.002	1.47±0.77	1.75±0.68	-1.175	0.248
	3.22±0.94	2.62±0.78	2.101	0.043	2.78±0.90	2.57±0.70	0.766	0.449	1.97±0.63	2.58±0.67	-2.8	0.008	1.54±0.77	1.76±0.72	-0.857	0.397
	3.43±0.87	2.74±0.71	2.595	0.014	3.03±0.83	2.70±0.73	1.271	0.212	1.79±0.48	2.48±0.62	-3.751	0.001	1.23±0.53	1.57±0.61	-1.819	0.078
	3.50±0.93	2.88±0.76	2.183	0.036	3.02±0.87	2.67±0.71	1.321	0.195	1.73±0.49	2.37±0.63	-3.398	0.002	1.24±0.60	1.58±0.57	-1.785	0.083
	3.31±0.86	2.75±0.71	2.135	0.04	3.10±0.85	2.76±0.79	1.227	0.228	1.80±0.44	2.39±0.60	-3.386	0.002	1.27±0.62	1.59±0.58	-1.581	0.123
	3.10±0.90	2.62±0.76	1.738	0.091	2.81±0.84	2.56±0.78	0.938	0.355	2.01±0.53	2.53±0.65	-2.618	0.013	1.59±0.85	1.81±0.72	-0.821	0.418
	3.02±0.80	2.57±0.70	1.789	0.083	3.28±0.95	2.99±0.91	0.94	0.354	1.93±0.52	2.49±0.75	-2.603	0.014	1.25±0.74	1.44±0.54	-0.856	0.398
	3.26±0.82	2.77±0.73	1.874	0.07	3.42±0.87	3.08±0.91	1.171	0.25	1.83±0.51	2.38±0.74	-2.59	0.014	0.94±0.43	1.24±0.39	-2.153	0.038
	3.44±0.86	2.93±0.75	1.895	0.067	3.63±0.84	3.19±0.93	1.486	0.146	1.65±0.44	2.21±0.66	-2.988	0.005	0.71±0.26	1.11±0.32	-4.141	0
	3.11±0.77	2.71±0.75	1.553	0.13	3.50±0.82	3.09±0.98	1.377	0.178	1.87±0.46	2.34±0.69	-2.417	0.021	0.99±0.40	1.34±0.43	-2.536	0.016
	2.88±0.81	2.53±0.72	1.344	0.188	3.37±0.84	3.01±0.95	1.217	0.232	2.00±0.50	2.43±0.67	-2.193	0.035	1.25±0.70	1.52±0.53	-1.31	0.199

	3.03±0.96	2.70±0.78	1.136	0.264	3.77±1.00	3.30±1.03	1.392	0.173	1.79±0.58	2.36±0.87	-2.293	0.028	0.88±0.61	1.12±0.40	-1.395	0.172
	3.19±0.94	2.80±0.77	1.361	0.182	3.87±0.95	3.34±1.03	1.61	0.117	1.70±0.56	2.30±0.83	-2.543	0.016	0.69±0.34	1.02±0.33	-2.959	0.006
	3.17±0.92	2.89±0.77	1.02	0.315	4.05±0.99	3.40±1.02	1.948	0.06	1.62±0.52	2.19±0.77	-2.58	0.014	0.62±0.24	1.00±0.33	-3.963	0
	2.94±0.86	2.74±0.78	0.739	0.465	4.08±1.02	3.48±1.14	1.654	0.107	1.73±0.56	2.19±0.74	-2.108	0.042	0.73±0.37	1.05±0.39	-2.554	0.015
	2.74±0.81	2.61±0.79	0.505	0.617	3.98±1.02	3.39±1.11	1.665	0.105	1.85±0.57	2.29±0.72	-2.019	0.051	0.92±0.58	1.20±0.43	-1.614	0.116
	3.07±0.96	2.81±0.78	0.891	0.379	4.12±1.05	3.43±1.05	1.972	0.057	1.63±0.55	2.22±0.81	-2.576	0.015	0.64±0.33	1.00±0.35	-3.109	0.004
	2.96±0.93	2.76±0.79	0.689	0.496	4.19±1.10	3.51±1.15	1.806	0.08	1.65±0.55	2.19±0.76	-2.403	0.022	0.68±0.36	1.02±0.39	-2.675	0.011
nontarget	3.46±0.91	2.96±0.80	1.772	0.085	2.82±0.76	2.46±0.67	1.51	0.14	1.78±0.52	2.44±0.56	-3.671	0.001	1.44±0.76	1.67±0.55	-1.007	0.321
	3.39±0.89	2.99±0.77	1.442	0.158	2.85±0.75	2.45±0.70	1.648	0.109	1.80±0.49	2.39±0.52	-3.508	0.001	1.46±0.80	1.69±0.57	-1	0.324
	3.18±0.86	2.76±0.75	1.543	0.132	2.82±0.84	2.52±0.75	1.112	0.274	1.96±0.58	2.53±0.63	-2.861	0.007	1.56±0.80	1.71±0.57	-0.655	0.517
	3.40±0.82	2.89±0.72	1.978	0.056	3.07±0.73	2.67±0.77	1.607	0.117	1.78±0.45	2.42±0.57	-3.716	0.001	1.22±0.56	1.53±0.47	-1.787	0.083
	3.48±0.84	3.02±0.79	1.676	0.103	3.03±0.76	2.60±0.74	1.745	0.09	1.74±0.46	2.34±0.56	-3.503	0.001	1.22±0.59	1.55±0.46	-1.863	0.071
	3.30±0.85	2.88±0.72	1.577	0.124	3.10±0.72	2.70±0.84	1.543	0.132	1.80±0.43	2.37±0.52	-3.575	0.001	1.27±0.65	1.55±0.53	-1.413	0.167
	3.07±0.88	2.75±0.72	1.171	0.25	2.82±0.74	2.50±0.83	1.238	0.224	2.02±0.51	2.52±0.57	-2.744	0.01	1.61±0.88	1.76±0.64	-0.602	0.551
	2.97±0.81	2.69±0.75	1.097	0.28	3.31±0.90	2.94±1.00	1.153	0.257	1.93±0.50	2.44±0.74	-2.446	0.02	1.27±0.73	1.42±0.51	-0.748	0.46
	3.21±0.82	2.84±0.80	1.368	0.18	3.45±0.81	3.04±0.98	1.365	0.181	1.85±0.53	2.35±0.73	-2.339	0.025	0.93±0.41	1.24±0.36	-2.434	0.02
	3.42±0.85	3.03±0.85	1.376	0.178	3.63±0.76	3.16±1.02	1.567	0.126	1.68±0.52	2.18±0.68	-2.477	0.018	0.68±0.25	1.10±0.32	-4.291	0
	3.08±0.77	2.79±0.81	1.097	0.28	3.48±0.73	3.04±1.08	1.435	0.16	1.91±0.50	2.33±0.68	-2.138	0.04	0.98±0.38	1.33±0.43	-2.577	0.014
	2.83±0.82	2.62±0.72	0.824	0.415	3.37±0.77	2.98±1.06	1.259	0.217	2.02±0.51	2.42±0.64	-2.073	0.046	1.26±0.69	1.48±0.54	-1.038	0.307
	2.91±0.94	2.75±0.85	0.526	0.602	3.84±0.93	3.25±1.09	1.744	0.09	1.83±0.58	2.35±0.84	-2.13	0.04	0.89±0.58	1.14±0.41	-1.487	0.146
	3.10±0.93	2.87±0.86	0.789	0.436	3.93±0.87	3.33±1.12	1.778	0.084	1.74±0.59	2.25±0.83	-2.13	0.041	0.67±0.30	1.02±0.35	-3.199	0.003
	3.11±0.91	2.95±0.87	0.555	0.583	4.07±0.89	3.36±1.09	2.114	0.042	1.67±0.56	2.17±0.78	-2.18	0.036	0.60±0.20	0.99±0.36	-4.096	0
	2.91±0.85	2.81±0.91	0.346	0.732	4.07±0.92	3.46±1.22	1.69	0.1	1.77±0.59	2.17±0.77	-1.749	0.089	0.71±0.34	1.03±0.42	-2.579	0.014
	2.68±0.81	2.68±0.87	0.021	0.983	3.98±0.93	3.34±1.20	1.775	0.085	1.92±0.59	2.30±0.74	-1.704	0.097	0.92±0.54	1.18±0.46	-1.552	0.13
	2.98±0.95	2.87±0.87	0.372	0.712	4.17±0.95	3.41±1.11	2.211	0.034	1.68±0.57	2.20±0.81	-2.256	0.031	0.63±0.30	1.00±0.37	-3.31	0.002
	2.88±0.90	2.82±0.90	0.175	0.862	4.21±0.98	3.48±1.21	2	0.054	1.72±0.58	2.18±0.77	-1.995	0.054	0.67±0.32	1.01±0.42	-2.734	0.01

Table S3. Four band cordance (unpaired t test).

Delta	Control	MDD	t	*p*	theta				alpha				beta			
**baseline**	**0.84±1.12**	**0.66±1.39**	**0.443**	**0.661**	**-1.48±0.84**	**-0.98±1.11**	**-1.53**	**0.135**	**-0.59±1.42**	**0.26±1.45**	**-1.789**	**0.083**	**0.76±1.36**	1.31±1.51	-1.147	0.259
	0.95±1.09	0.71±1.48	0.574	0.57	-1.38±0.76	-0.99±1.07	-1.25	0.22	-0.61±1.37	-0.15±1.38	-1.004	0.323	0.56±1.31	0.96±1.27	-0.928	0.36
	0.29±1.00	0.17±1.10	0.347	0.731	-1.42±0.70	-0.58±1.08	-2.793	0.009	0.04±1.16	0.89±1.44	-1.952	0.059	1.45±1.16	1.52±1.41	-0.171	0.866
	0.25±0.80	0.13±0.95	0.407	0.687	-1.16±0.66	-0.80±0.81	-1.486	0.147	-0.59±0.96	0.04±1.15	-1.79	0.082	0.15±1.01	0.57±1.11	-1.167	0.251
	0.54±0.97	0.26±1.10	0.802	0.428	-1.26±0.85	-1.11±0.94	-0.502	0.619	-0.91±1.10	-0.53±1.03	-1.078	0.289	-0.04±0.87	0.52±1.08	-1.717	0.095
	0.24±0.74	0.10±1.03	0.478	0.636	-0.99±0.51	-0.85±0.73	-0.668	0.508	-0.62±0.70	-0.51±0.96	-0.396	0.695	0.16±0.71	0.15±0.98	0.035	0.972
	0.39±0.91	0.10±1.07	0.864	0.393	-1.13±0.56	-0.72±1.05	-1.487	0.146	-0.04±1.00	0.39±1.52	-0.988	0.33	1.46±1.43	1.03±1.36	0.917	0.365
	-0.36±0.67	-0.48±0.81	0.482	0.633	-0.32±0.78	0.06±0.85	-1.407	0.168	0.37±1.02	0.81±0.97	-1.321	0.195	0.89±1.14	0.48±1.31	1.014	0.318
	-0.57±0.56	-0.54±0.68	-0.127	0.9	-0.44±0.45	-0.03±0.92	-1.691	0.1	-0.41±0.69	-0.08±1.22	-0.98	0.334	-0.53±0.79	-0.53±0.75	-0.004	0.997
	-0.37±0.76	-0.28±0.86	-0.331	0.743	-0.17±0.69	-0.35±0.95	0.651	0.519	-0.76±0.65	-1.14±0.89	1.446	0.157	-1.31±1.13	-1.12±0.83	-0.578	0.567
	-0.64±0.48	-0.76±0.76	0.551	0.585	-0.16±0.45	-0.44±0.77	1.319	0.196	-0.01±0.74	-0.59±0.80	2.24	0.032	-0.03±1.04	-0.48±0.93	1.379	0.177
	-0.47±0.64	-0.20±0.94	-1.017	0.316	0.05±0.89	0.17±0.70	-0.468	0.643	0.58±1.14	0.23±1.13	0.916	0.366	1.00±1.09	0.37±1.02	1.784	0.083
	-0.43±1.23	-0.38±0.98	-0.136	0.893	0.78±0.98	0.72±1.04	0.183	0.856	0.35±1.07	0.41±0.97	-0.204	0.84	-0.39±0.97	-0.59±0.70	0.697	0.491
	-0.16±0.95	-0.17±0.81	0.018	0.985	0.91±0.58	0.69±0.83	0.939	0.354	0.20±0.96	-0.09±1.03	0.901	0.374	-1.03±0.69	-1.06±0.65	0.151	0.88
	0.02±0.72	0.11±0.93	-0.312	0.757	1.29±0.61	0.77±0.78	2.208	0.034	0.15±0.91	-0.33±1.05	1.462	0.153	-1.12±0.83	-0.92±0.92	-0.685	0.498
	-0.21±0.77	0.15±1.02	-1.213	0.233	1.58±0.87	1.05±1.20	1.514	0.139	0.76±0.86	-0.16±1.27	2.552	0.015	-0.54±0.84	-0.67±0.94	0.445	0.659
	-0.39±0.92	-0.01±1.06	-1.147	0.26	1.68±1.02	1.10±1.28	1.525	0.136	0.99±1.01	0.51±1.29	1.243	0.223	0.22±1.18	-0.07±1.24	0.719	0.477
	0.11±1.01	0.15±0.91	-0.103	0.919	1.71±0.63	1.06±0.95	2.432	0.02	0.41±0.90	0.03±1.21	1.095	0.281	-1.01±0.67	-0.84±0.93	-0.624	0.537
	-0.04±0.86	0.28±1.24	-0.9	0.374	1.91±0.74	1.22±1.35	1.888	0.068	0.70±0.80	0.01±1.28	1.937	0.061	-0.67±0.83	-0.64±1.04	-0.093	0.926
target	0.54±1.58	0.24±1.45	0.59	0.559	-1.44±0.81	-1.13±0.84	-1.146	0.26	-0.19±1.52	0.54±1.08	-1.666	0.105	1.16±1.44	1.37±1.30	-0.476	0.637
	0.24±1.40	0.24±1.27	-0.01	0.992	-1.40±0.83	-1.26±0.65	-0.572	0.571	-0.13±1.48	0.16±1.18	-0.668	0.509	1.09±1.63	1.34±1.10	-0.553	0.584
	-0.14±1.20	-0.19±1.07	0.113	0.911	-1.50±0.59	-0.84±0.91	-2.582	0.014	0.58±1.48	1.05±1.02	-1.12	0.271	1.83±1.28	1.66±1.11	0.417	0.679
	0.12±0.76	-0.11±0.95	0.825	0.415	-1.18±0.65	-0.82±0.93	-1.341	0.189	-0.40±1.00	0.35±0.98	-2.26	0.03	0.37±1.14	0.59±0.91	-0.636	0.529
	0.34±1.03	0.01±1.14	0.894	0.378	-1.19±0.91	-1.21±0.71	0.075	0.941	-0.80±1.19	-0.47±0.87	-0.937	0.355	0.14±1.19	0.42±0.86	-0.783	0.439
	-0.14±0.69	-0.21±0.87	0.282	0.78	-1.00±0.68	-0.91±0.46	-0.482	0.633	-0.37±0.97	-0.16±1.02	-0.643	0.525	0.36±1.10	0.59±1.06	-0.631	0.533
	-0.36±1.02	-0.37±0.88	0.037	0.971	-1.32±0.70	-1.12±0.85	-0.786	0.437	0.85±1.31	0.77±1.80	0.158	0.876	1.99±1.15	1.74±1.57	0.543	0.591
	-0.54±0.54	-0.38±0.69	-0.776	0.443	-0.45±0.70	0.18±0.82	-2.482	0.018	0.46±0.72	0.81±1.24	-1.039	0.306	0.82±1.17	0.53±1.17	0.74	0.464
	-0.22±0.82	-0.31±0.65	0.339	0.737	-0.49±0.53	-0.13±0.93	-1.443	0.158	-0.41±0.74	-0.26±1.13	-0.448	0.657	-0.64±0.72	-0.74±0.65	0.457	0.651
	0.12±1.17	-0.01±0.81	0.379	0.707	-0.23±0.72	-0.10±1.03	-0.438	0.664	-1.27±0.79	-1.12±1.12	-0.445	0.659	-1.66±0.59	-1.37±0.77	-1.285	0.208
	-0.56±0.86	-0.76±0.56	0.822	0.417	-0.30±0.46	-0.46±0.86	0.69	0.495	-0.13±0.74	-0.57±1.10	1.402	0.17	-0.31±0.87	-0.44±0.94	0.414	0.682
	-0.76±0.67	-0.51±0.68	-1.118	0.271	-0.06±0.62	0.07±0.64	-0.632	0.532	1.05±1.22	0.58±1.32	1.101	0.279	1.25±1.42	1.00±1.18	0.572	0.571

	-0.02±1.32	-0.01±0.93	-0.017	0.987	0.79±1.06	0.79±0.95	0.025	0.98	0.15±0.86	0.09±1.23	0.164	0.871	-0.58±1.17	-0.78±0.80	0.604	0.55
	0.37±1.15	0.23±0.71	0.425	0.674	0.86±0.71	0.78±0.49	0.369	0.715	-0.24±0.84	-0.18±1.05	-0.184	0.855	-1.35±0.68	-1.26±0.63	-0.411	0.683
	0.53±0.93	0.57±0.80	-0.145	0.885	1.44±0.69	0.88±0.81	2.247	0.031	-0.38±0.88	-0.63±0.78	0.906	0.372	-1.48±0.61	-1.34±0.50	-0.768	0.448
	0.00±0.82	0.33±0.88	-1.163	0.253	1.68±0.81	1.27±0.97	1.379	0.177	0.22±1.21	-0.44±1.04	1.764	0.087	-0.89±0.74	-0.90±0.72	0.04	0.968
	-0.37±0.69	0.04±1.04	-1.403	0.17	1.78±0.91	1.23±1.21	1.537	0.134	0.96±1.14	0.11±1.10	2.281	0.029	0.18±1.26	-0.24±1.01	1.104	0.277
	0.54±0.97	0.65±0.85	-0.373	0.711	1.88±1.01	1.29±0.92	1.818	0.078	-0.10±0.91	-0.26±1.07	0.479	0.635	-1.33±0.71	-1.18±0.66	-0.677	0.503
	0.33±0.89	0.54±1.02	-0.664	0.511	2.14±0.88	1.47±1.32	1.779	0.084	0.17±0.95	-0.35±1.03	1.57	0.126	-0.95±0.77	-1.01±0.69	0.237	0.814
nontarget	0.60±1.35	0.64±1.71	-0.078	0.939	-1.51±0.78	-1.19±0.87	-1.17	0.25	-0.38±1.40	0.49±1.17	-2.018	0.051	1.09±1.37	1.41±1.35	-0.722	0.475
	0.31±1.21	0.57±1.55	-0.578	0.567	-1.47±0.73	-1.42±0.60	-0.249	0.805	-0.33±1.43	0.11±1.31	-0.977	0.336	0.99±1.53	1.28±1.11	-0.654	0.517
	-0.14±1.07	0.08±1.23	-0.572	0.571	-1.49±0.66	-0.93±0.94	-2.051	0.048	0.41±1.52	1.04±1.02	-1.442	0.158	1.88±1.33	1.82±1.14	0.144	0.886
	0.15±0.77	0.12±1.12	0.088	0.93	-1.18±0.69	-0.85±0.91	-1.245	0.222	-0.54±0.84	0.25±0.95	-2.63	0.013	0.31±1.09	0.66±0.96	-1.002	0.323
	0.40±0.91	0.29±1.37	0.286	0.777	-1.26±0.84	-1.28±0.73	0.081	0.936	-0.85±1.04	-0.42±0.96	-1.274	0.211	0.02±1.10	0.49±0.97	-1.344	0.188
	-0.06±0.76	-0.11±1.01	0.153	0.879	-1.06±0.57	-1.03±0.40	-0.207	0.837	-0.53±1.01	-0.18±0.98	-1.061	0.296	0.30±1.05	0.49±1.04	-0.547	0.588
	-0.36±0.95	-0.27±1.02	-0.265	0.792	-1.40±0.62	-1.28±0.93	-0.459	0.649	0.72±1.44	0.80±1.90	-0.148	0.883	2.00±1.10	1.72±1.71	0.586	0.562
	-0.52±0.56	-0.39±0.77	-0.558	0.581	-0.36±0.76	0.02±0.90	-1.376	0.178	0.42±0.83	0.65±1.13	-0.683	0.499	0.97±1.22	0.58±1.14	0.979	0.335
	-0.24±0.66	-0.42±0.63	0.84	0.407	-0.43±0.50	-0.06±0.98	-1.416	0.166	-0.36±0.85	-0.23±1.30	-0.368	0.716	-0.69±0.78	-0.59±0.72	-0.382	0.705
	0.14±0.99	-0.04±0.81	0.598	0.554	-0.24±0.67	0.07±1.23	-0.934	0.357	-1.20±0.91	-1.06±1.41	-0.362	0.72	-1.75±0.60	-1.33±0.92	-1.611	0.116
	-0.52±0.80	-0.85±0.64	1.381	0.176	-0.34±0.43	-0.43±0.93	0.4	0.692	-0.02±0.74	-0.38±1.32	1.005	0.322	-0.28±0.86	-0.33±1.16	0.149	0.882
	-0.76±0.63	-0.64±0.77	-0.532	0.598	-0.12±0.61	0.08±0.64	-0.94	0.354	0.97±1.17	0.65±1.47	0.713	0.481	1.31±1.24	0.90±1.47	0.917	0.365
	-0.19±1.28	-0.26±1.10	0.175	0.862	0.95±1.03	0.74±1.10	0.6	0.552	0.27±0.85	0.09±1.20	0.522	0.605	-0.45±1.15	-0.76±0.84	0.922	0.363
	0.25±1.08	0.07±0.80	0.567	0.574	0.97±0.65	0.95±0.42	0.121	0.905	-0.16±0.94	-0.31±1.00	0.482	0.633	-1.37±0.60	-1.33±0.74	-0.144	0.887
	0.49±0.82	0.40±0.91	0.306	0.762	1.45±0.63	1.01±0.79	1.875	0.069	-0.25±0.89	-0.63±0.83	1.305	0.201	-1.54±0.57	-1.37±0.58	-0.887	0.381
	0.09±0.56	0.20±1.04	-0.393	0.697	1.65±0.75	1.44±0.91	0.751	0.458	0.32±1.18	-0.42±1.09	1.942	0.06	-0.87±0.76	-1.00±0.71	0.527	0.601
	-0.34±0.67	-0.16±1.27	-0.536	0.596	1.74±0.86	1.23±1.23	1.427	0.163	1.16±1.16	0.26±1.27	2.227	0.033	0.31±1.20	-0.23±1.10	1.414	0.166
	0.46±1.00	0.41±0.99	0.135	0.894	1.98±0.97	1.39±0.95	1.85	0.073	0.03±0.90	-0.33±1.09	1.1	0.279	-1.30±0.64	-1.27±0.77	-0.124	0.902
	0.25±0.85	0.36±1.18	-0.311	0.757	2.11±0.88	1.54±1.29	1.535	0.134	0.31±0.96	-0.38±1.09	2.019	0.051	-0.93±0.79	-1.12±0.65	0.791	0.435
